# Pan-plastome approach empowers the assessment of genetic variation in cultivated *Capsicum* species

**DOI:** 10.1038/s41438-019-0191-x

**Published:** 2019-09-07

**Authors:** Mahmoud Magdy, Lijun Ou, Huiyang Yu, Rong Chen, Yuhong Zhou, Heba Hassan, Bihong Feng, Nathan Taitano, Esther van der Knaap, Xuexiao Zou, Feng Li, Bo Ouyang

**Affiliations:** 1 Key Laboratory of Horticultural Plant Biology (Ministry of Education), Huazhong Agricultural University, 430070 Wuhan, China; 2 Genetics Department, Faculty of Agriculture, Ain Shams University, Cairo, 11241 Egypt; 3 College of Horticulture and Landscape, Hunan Agricultural University, 410128 Changsha, China; 4 College of Agriculture, Guangxi University, 530004 Nanning, China; 5 Department of Horticulture, College of Agriculture & Environmental Sciences, University of Georgia, Athens, GA 30602 USA

**Keywords:** Comparative genomics, Phylogenomics, Agricultural genetics, Genetic markers

## Abstract

Pepper species (*Capsicum* spp.) are widely used as food, spice, decoration, and medicine. Despite the recent old-world culinary impact, more than 50 commercially recognized pod types have been recorded worldwide from three taxonomic complexes (A, B, and P). The current study aimed to apply a pan-plastome approach to resolve the plastomic boundaries among those complexes and identify effective loci for the taxonomical resolution and molecular identification of the studied species/varieties. High-resolution pan-plastomes of five species and two varieties were assembled and compared from 321 accessions. Phyloplastomic and network analyses clarified the taxonomic position of the studied species/varieties and revealed a pronounced number of accessions to be the rare and endemic species, *C. galapagoense*, that were mistakenly labeled as *C. annuum* var. *glabriusculum* among others. Similarly, some NCBI-deposited plastomes were clustered differently from their labels. The *rpl*23-*trn*I intergenic spacer contained a 44 bp tandem repeat that, in addition to other InDels, was capable of discriminating the investigated *Capsicum* species/varieties. The *rps*16-*trn*Q/*rbc*L-*acc*D/*ycf*3-*trn*S gene set was determined to be sufficiently polymorphic to retrieve the complete phyloplastomic signal among the studied *Capsicum* spp. The pan-plastome approach was shown to be useful in resolving the taxonomical complexes, settling the incomplete lineage sorting conflict and developing a molecular marker set for *Capsicum* spp. identification.

## Introduction

Peppers are a major global crop and one of the top five most abundant vegetables in China^1^ that are cultivated as food, as a source of spices, and for pharmaceutical and ornamental purposes^2^. Currently, the *Capsicum* genus consists of ~32 wild and five cultivated species^3^. Pepper fruits are the commodity of pepper plants. Therefore, fruit morphology, flavor, and pungency are the most economically important characteristics of *Capsicum*. Major genetic variation has been detected in fruit-based morphologies, which has resulted in more than 50 commercially recognized pod types^4^. Given the continuing confusion regarding the best method to identify the traditionally recognized domesticated species of *Capsicum*, several key features have been proposed (e.g., seed color, corolla color and pattern, filament color, and the number of flowers per node) to distinguish the five cultivated *Capsicum* species^5^. Those key features have been widely evaluated in phenotypic, chromosomal, and hybridization studies^6,7^. The cultivated species are grouped into three complexes, each comprising species of possible fertile hybrids: the annuum complex, comprising the species *C. annuum* L. (vars. *glabriusculum* (Dunal) Heiser & Pickersgill and *annuum*), *C. frutescens* L., *C. chinense* Jacq., and *C. galapagoense* Hunz.; the baccatum complex, composed of the species *C. chacoense* Hunz. and *C. baccatum* L. (vars. *baccatum* and *pendulum* (Willd.) Eshbaugh), *C. praetermissum* Heiser & P.G. Sm. and *C. tovarii* Eshbaugh, P.G. Sm. & Nickrent, and the pubescent complex, consisting of the species *C. cardenasii*, *C. eximium* and *C. pubescens* Ruiz & Pav^8–11^. The triple origin of the domesticated *Capsicum* species was confirmed by both chromosomal karyotyping^9^ and molecular phylogenetic studies^8^. However, these studies contrast each other; Moscone et al.^9^ were able to provide sufficient information to separate the unclear annuum complex species of the genus, while a clear distinction at the molecular level has still not been resolved. The *C. annuum* complex was characterized as the most derived clade. However, some interspecific relationships within cultivated *Capsicum* complexes are unresolved, and uncertainties remain mostly due to insufficient species sampling not only for the aforementioned *Capsicum* complexes but also for the whole genus^10^.

In higher plants, chloroplast genome sequences contain loci that are considered key loci when resolving taxonomical complexes and/or DNA barcoding (e.g., *mat*k and *rbc*L)^12^. Unfortunately, most of the commonly applied chloroplast loci often lack variation in very closely related species or among varieties (e.g., *Capsicum* spp.^8^). With the advent of next‐generation sequencing (NGS) technologies, plastome‐scale data may be easily assembled for multipurpose tasks^13^. The *Capsicum* spp. chloroplast genome is a single circular chromosome with a quadripartite structure comprising a pair of inverted repeats (IRs) separated by two single-copy regions (long “LSC” and short “SSC”), which is a typical structure of chloroplast genomes^11,14^. The high abundance level and conserved characteristics of the plastome enables the use of NGS-based methods in the genus *Capsicum* to infer the genotypes of whole plastomes with high accuracy using a mapping approach and/or combined with de novo assembly. Previous studies on *Capsicum* spp. plastomes have performed a family-based comparative analysis^14^, reported the full plastome sequence^15,16^ or performed an interspecies/variety comparative analysis to develop molecular markers^11^. The main interspecific variations were demonstrated by contractions and expansions of the IR region and intergenic spacers (IGSs), and by the nucleotide diversity within specific CDSs (e.g., *acc*D, *ndh*B, *rpl*20, *ycf*1, and *ycf*2)^11,14^. However, the common drawback among the previous reports was the number of accessions per species. Multiple numbers per species/variety would help in (a) avoiding unclear clustering of single accessions, (b) effectively assessing inter- and intraspecific variation at the plastome level, and (c) developing efficient markers to ensure the correct identification of the assembled plastome.

In parallel with the creation of the nuclear pan‐genome of cultivated pepper from 383 diverse lines representing four species (*C. annuum*, *C. chinense*, *C. frutescens*, and *C. baccatum*)^17^, the whole-genome sequencing (WGS) paired‐end reads were used to assemble the corresponding plastomes to (a) enrich, update and improve the growing database of *Capsicum* genomics, (b) improve the phylogenetic resolution of the defined species complexes, and (c) develop reproducible molecular markers for DNA barcoding, population-based studies, phylogeography, and domestication genetics.

## Results

### Plastid genome structure and organization

**Fig. 1:**
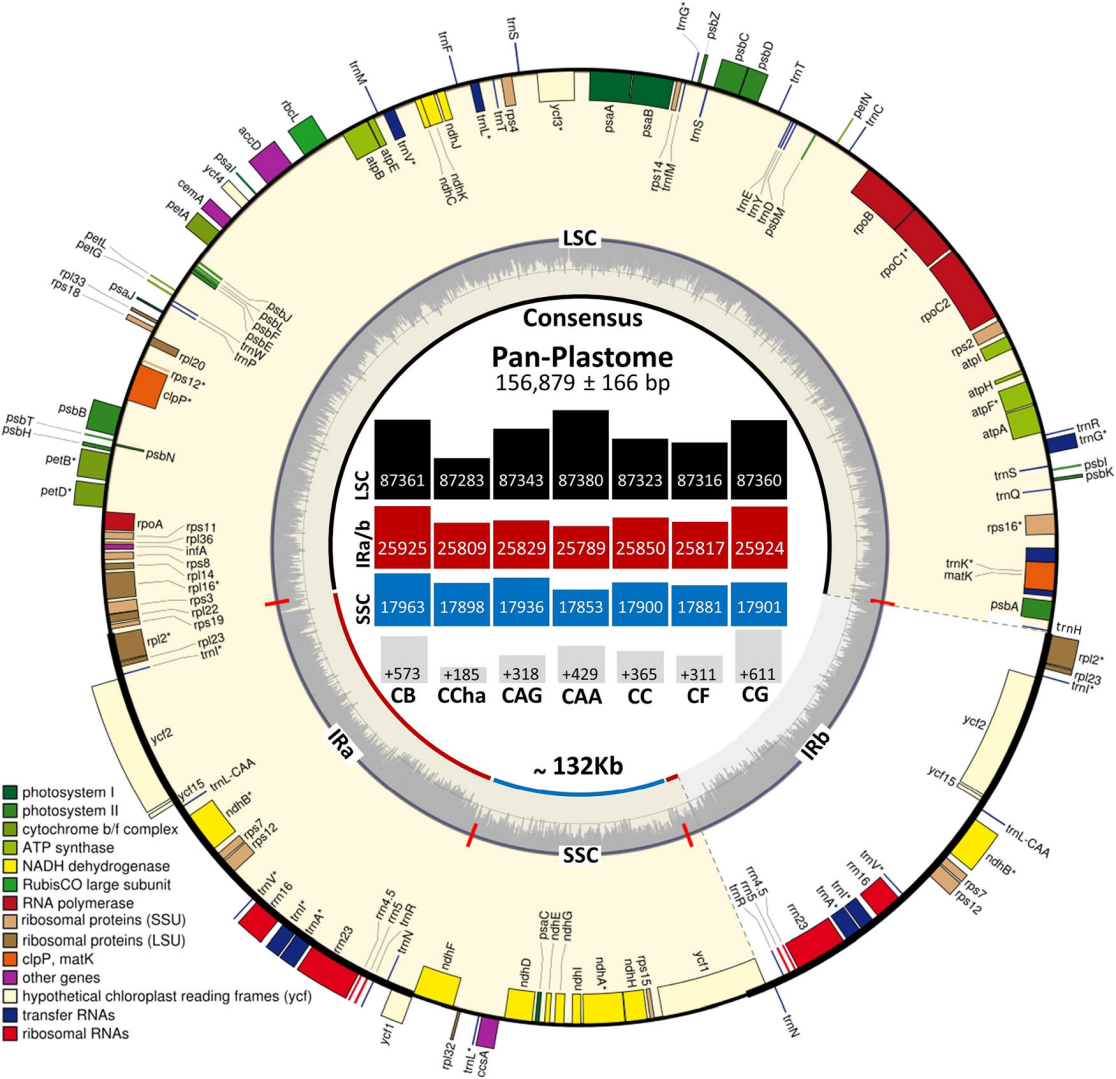
The complete structural chloroplast genome based on the consensus pan-plastome (average size 156,879 ± 166 bp). The inner genes of the outer circle are transcribed counterclockwise while the outer genes are transcribed clockwise; genes with introns were marked with (*). The dashed area in the inner circle indicates the GC content and is separated into regions as follows: LSC large single-copy, SSC short single-copy, and IR inverted repeats (**a**) and (**b**) at the outer border, while the inner border indicates the trimmed pan-plastome used for further analysis (the IRb region trimmed to the least that the *ycf*1 gene shared between the SSC and IRb regions was fully annotated). The size reached ~132 kb. Within the inner circle, the average sizes of LSC (black), IR (red; either **a** or **b**) and SSC (blue) regions are indicated, while the exact size of the alignment above 132 kb (gray) is indicated for each species/variety

From 448 samples, a total of 321 complete chloroplast genomes were successfully de novo assembled and analyzed. The length varied between 156,616–157,641 bp with Med = 156,817 bp and x̅ = 156,879 ± 166 bp (Supplementary Table 1). The structure of the genomes was a typical quadripartite circular molecule composed of one LSC region of 87,216–87,901 bp (Med = 87,380 bp and x̅ = 87,371 ± 44 bp), one SSC region of 17,840–17,996 bp (Med = 17,853 bp and x̅ = 873 ± 39 bp), and a pair of IR regions of 25,735–26,160 bp (Med = 25,792 bp and x̅ = 25,817 ± 66 bp; Fig. 1; Supplementary Table 1). In terms of synteny and gene number, both were highly conserved in the assembled accessions and in accordance with the reference accession (*C. annuum*; GenBank: JX270811)^14^. In general, the plastome consists of 79 protein-coding genes (six repeated/IR), 30 tRNA genes (seven repeated/IR), and 8 rRNA genes (four repeated/IR). Among the detected genes, 20 genes contained introns, 12 CDSs (*rps*16, *atp*F, *rpo*C1, *ycf*3, *rps*18, *rps*12, *rpl*2, two copies of *ycf*2, two copies of *ndh*B, and *ndh*A), and eight tRNA genes (*trn*K-UUU, *trn*G-GCC, *trn*L-UAA, *trn*V-UAC, two copies of *trn*I-GAU, and two copies of *trn*A-UGC) (Fig. 1; Supplementary Table 2).

### Plastome polymorphism

The full-length plastomes and the 23 cp genomes deposited in the GenBank database were aligned. When the fixed gaps (sites showing 100% gaps) were excluded, the total alignment length was 162,057 bp (InDels = 7371 sites). A total of 154,219 bp were invariable, and 756 were variable. Among the variable sites, 345 were singletons (included as unique three-variant sites at *psb*C-*trn*S), and 411 were parsimony informative (406 sites were two-variant sites, and five were three-variant sites, namely, the *trn*K intron (V), *trn*S (B), *rpl*36 (D), *rpl*32-*trn*L (D), and *ycf*1 (B)). The trimmed pan-plastome alignment length was 133,524 bp (InDels included). A total of 130,497 sites were identical, with 3027 InDels forming 206 nonoverlapping InDel events and 712 sites of non-gapped variants. Among the variant sites, 324 were singletons, and 383 were parsimony informative (including the previously mentioned two- and three-variant sites). Based on the 100% similarity index, when gaps of 99% were included or excluded, the accessions were collapsed to a final set of 212 haplotypes (Hd = 0.98) or 85 (Hd = 0.90), respectively.

### Phyloplastomics and species delimitation

**Fig. 2:**
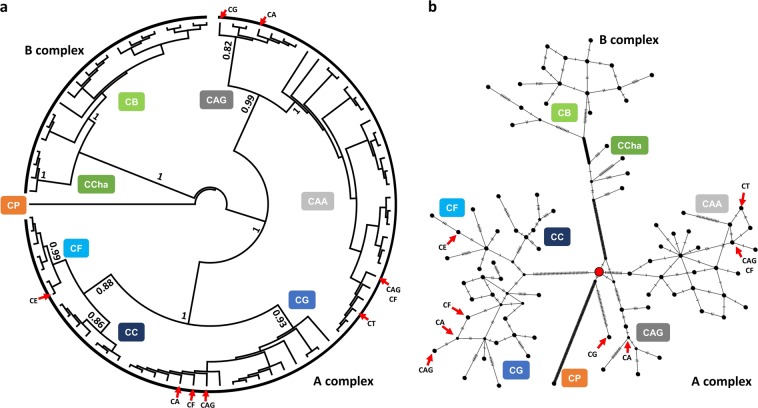
Phylogenomic analysis of the 85 haplotypes based on (**a**) BI methods and (**b**) a minimum spanning network. For both parts of the figure, CP was used as an outgroup/root; each species is a separate clade from other species. The A complex is formed by CAA, CAG, CC, CF, and CG, where the phylogenetic signal is equal between the BI tree and the network for the CC, CF, and CG species. The B complex is formed by CCha and CB, while it clearly resolved the position of CCha in between the CB and the A complex species. The red node represents the most common ancestral connection among the groups

Based on the Bayesian inference (BI) tree generated for both the non-gapped haplotypes (Fig. 2a) and the gapped haplotypes (Supplementary Fig. 2a) rooted by the *C. pubescens* (CP) species, seven highly supported monophyletic groups were defined (mean bootstrap support >0.80). According to the literature and based on the reference plastomes included in the analysis, the groups were separated into two major clades. One was named the A complex, which was defined by two highly supported subclades. The first subclade showed the two *C. annuum* (CA) varieties, the var. *glabriusculum* (CAG) and var. *annuum* (CAA). The second subclade included two minor clades, one including two clusters, one cluster representing the *C. frutescens* (CF) species and another cluster representing the *C. chinense* (CC) species, and a highly supported minor clade named *C. galapagoense* (CG) species. The other major clade, named the B complex, was defined by two subclades, one for *C. chacoense* (CCha) species and the other for the *C. baccatum* (CB) species (including the different varieties), and both were highly supported (Fig. 2a). The haplotype network showed a similar pattern of the relationship as the BI tree. The CC, CF, and CG groups were more clustered at a distance from the CAA and CAG groups. The network clearly inferred the position of the CCha between the A complex and the CB group, and the unknown most common recent ancestral connection was shared between CFG (CC, CF, and CG), CCha, CAA, CAG, and CP (Fig. 2b; Supplementary Fig. 2b).

Based on the BI tree and the haplotype network, some accessions and referenced plastomes were not grouped according to their herbarium labels. The case was present in the A complex major clade, while in the CB clade, the incongruence of the clustering of the referenced plastomes was the reason for not defining the CB varieties correctly. Due to the presence of CG accessions that had never been labeled and the inconsistency between the accession clustering and their original labels, interplastome recombination and species delimitation analyses were analyzed for each cluster inferred by the BI tree. No sign of recombination within each group was found in the studied accessions; however, a referenced genome (CG; GenBank: MH559323)^11^ was determined to be recombinant and further excluded. Each of the seven groups was found to be monophyletic, with an intradistance less than the interdistance to the closest clade (Intra/interdistance <1). The A complex was more diverse (0.002) than the B complex (0.001). CAA and CG were the most diverse species/variety, followed by CB, CC, CAG and finally CF and CCha. Based on the most recent common ancestor (MRCA)-tips, CP was defined as the most distant species, followed by both CCha and CF in the parallel complexes and CAG for the CA subclade (Table 1).

**Table 1:** Species delimitation based on the Bayesian inference tree

Species/variety	Closest species/variety	Monophyletic?	Intradistance	Interdistance to closest	Intra/interdistance	Av (MRCA-tips)
CP	CAG	Yes	0.000	0.222	0.00	0.0000
CCha	CB	Yes	0.019	0.177	0.11	0.0119
CB	CCha	Yes	0.013	0.177	0.07	0.0140
CC	CF	Yes	0.013	0.032	0.39	0.0072
CF	CC	Yes	0.021	0.032	0.64	0.0133
CG	CF	Yes	0.021	0.037	0.56	0.0146
CAG	CAA	Yes	0.012	0.086	0.14	0.0108
CAA	CAG	Yes	0.052	0.086	0.60	0.0261
A complex	B complex	Yes	0.076	0.216	0.35	0.0657
B complex	A complex	Yes	0.042	0.216	0.20	0.0883

The monophyletic status, the intraspecific distance (Intradistance), the interspecific distance to the closest species/variety (Interdistance), the intraspecific distance relative to the interspecific distance (Intra/Inter), and the average most recent common ancestor tips (Av MRCA-tips) were estimated for each defined group and the two major clusters (A and B complexes)

### Genetic diversity assessment

#### Intraspecies/variety variation

Each species/variety group was labeled as delimited, and then each group was separated and realigned. Except for *C. pubescens*, the number of mutations varied from 52 for CB and CG to 13 for CC, and the number of haplotypes was the highest in CG (28) and the lowest in CCha (4). All of the recorded haplotypes showed high genetic diversity (>0.8); however, for the CAA group (238 accessions), the number of mutations (19) exhibited the second highest haplotype number (24) but was the group with the lowest haplotype diversity (0.8). The number of simple sequence repeats (SSRs) was assessed and ranged between 371 (CB) and 360 (CF). One AT repeat unit located in the *rps*16-*trn*Q IGS was polymorphic for all the species belonging to the A complex, except for the CC species, with variable lengths ranging from 101 bp in CAA to 30 bp in CAG. The AAATT repeat unit located in the *psa*A-*ycf*3 IGS was found to be polymorphic within CAA and CF samples, and the AAT unit located in the *ycf*1 CDS was found to be polymorphic within CAG and CG. CB uniquely showed a polymorphic AAT SSR at *rbc*L-*acc*D, while CG showed two AAT repeat units located in the same loci (*ndh*C-*trn*V) at two separate InDel events. Neither CCha nor CC showed any interspecific polymorphism at the SSR level (Supplementary Table 3).

#### Pan-plastome and interspecies/variety diversity

The pan-plastomes generated for each species/variety were realigned. The alignment length of 134,244 bp was recorded, and 3,234 bp were InDel sites forming 144 InDel events and 350 non-gapped variant sites with 354 Eta. The InDels were neutral, as Tajima D was not significant.

#### Large InDels, tandem repeats, and SSRs

The *C. annuum* plastome, when compared to plastomes of other members of the Solanaceae family, showed two distinct features: frequent tandem repeat sequences and large InDels, including insertions in *acc*D and *rpl*20 gene sequences^14^. Gaps, when present, can be caused by insertion/deletion events, tandem repeats or simple sequence repeats. On the one hand, the changes in loci sizes were investigated, each CDS, intron, and IGS were measured in bp for each species/variety relative to CP, and the deviation average for each locus was estimated as an indicator for intraspecific length variation. Three CDS genes (*acc*D, *rpl*20, and *ycf*1) showed a suitable amount of length variation for PCR-based analysis (deviation average >25 bp). Similarly, six IGSs (*rpl*23-*trn*I, *rps*19-*rpl*2, *rps*16-*trn*Q, *rpl*32-*trn*L, *trn*L-*trn*F, and *psa*A-*ycf*3, ordered by deviation average from 90 bp to 25 bp) showed variable lengths among the pan-plastomes (Supplementary Fig. 3a). The IGSs *rps*9-*rpl*2 and *rpl*32-*tnr*L were found to be polymorphic due to the unique InDel for CAA (deletion) and CF (insertion), respectively (Supplementary Fig. 3a).

On the other hand, the changes in each locus were subject to tandem repeat and SSR analysis to investigate the causes of such differences. Tandem repeats were identified for each pan-plastome. A total of 30 perfect tandem repeats were detected, 14 of which were polymorphic. The highest number of repeats was detected in *acc*D, varying from 6 to 13 copies of 18 bp unit, followed by *rpl*23-*trn*I, which recorded 3 to 6 copies of a 44 bp unit. In addition to the two polymorphic repeats in *rps*16-*trn*Q, *trn*S-*trn*G, and *trn*L-*trn*F, one polymorphic repeat was identified in the *trn*G intron, the *ycf*3 intron, *acc*D-*psa*I, and *cem*A-*pet*A (Supplementary Table 4).

Among all species/varieties, none of the detected repeats showed equal frequency among the pan-plastomes, which was reflected in several variable loci when the seven pan-plastomes were compared. The stacked column plot for the repeats along the plastomes visually revealed those variable sites. Some sites with major differences in repeat frequency were identified among the seven pan-plastomes and with a suitable size for PCR-based analysis, namely, *rps*16-*trn*Q, *psa*A-*ycf*3, and *ndh*C-*trn*V, which were all previously shown to be polymorphic within some of the species/variety (Supplementary Fig. 3b). Alternatively, the Phobos plugin in Geneious was used to detect the polymorphic SSR loci, and four loci located in *rps*16-*trn*Q, *psa*A-*ycf*3, the *ycf*3 intron, and *ycf*1 were detected.

#### Interspecies/variety SNP-based variations

**Fig. 3:**
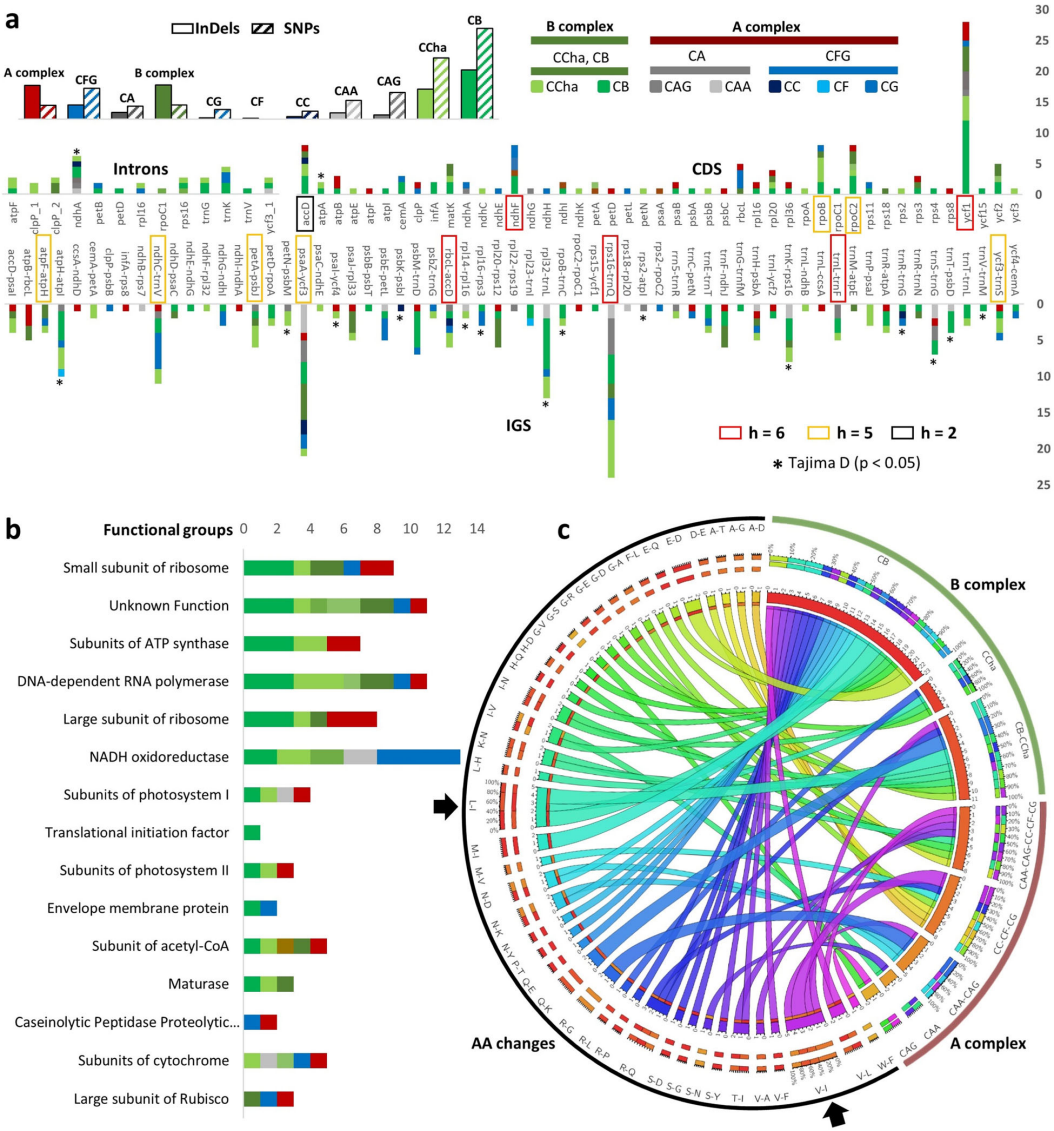
SNP-based genetic variation in each cultivated *Capsicum* species/variety. **a** Collective stacked column plots for the number of SNP-based polymorphic sites and InDel-based polymorphic sites recorded for each species/variety/group are shown; unique and common SNP counts for every intron and CDS (columns up) and IGS (columns down) per species/variety/group, unneutral loci were marked by an asterisk (*) while neutral highly polymorphic loci were boxed in a colored box (red: haplotypes number h = 6, orange: h = 5, and black: h = 2). **b** Common and unique SNPs recorded for each functional group per species/variety/group, where the NADH accumulated the highest number of SNPs. **c** Circos plot shows the number of unique and common amino-acid changes per species/variety/group; where L > I and V > I (indicated by black arrows) are the two most frequent common amino-acid changes for the B complex and A complex, respectively

After masking the ambiguities in the pan-alignment, interspecies/variety diversity was assessed using the CP plastome as a reference. The A complex, B complex, CAA/CAG (CA) subclade, and CC/CF/CG (CFG) subclade were each considered as a “*Capsicum* group” for variation assessment in addition to the seven *Capsicum* species/varieties. Among the species/variety, the number of polymorphic sites ranged from 2–332 sites (0–215 InDels and 2–117 SNPs), the minimum values were recorded for the CF and the maximum for the CB. The common polymorphic sites among the defined groups ranged between 16 and 81 sites, and the number of InDels was lower than the number of SNPs for the A and B complex groups (34 and 33 sites InDels; 81 and 80 SNPs, respectively). Within the A complex, the number of InDels was higher than the number of SNPs for the CA and CFG groups (31 and 73 InDels; 16 and 34 SNPs, respectively; Fig. 3a; Supplementary Fig. 4).

##### Intergenic spacers and introns

Among intergenic spacers, 243 SNPs were recorded from 63 IGSs. The number of SNPs ranged from 1–24 sites, and the maximum number of SNPs was found in *rps*16-*trn*Q, followed by *psa*A-*ycf*3 (21), *rpl*32-*trn*L (13), *ndh*C-*trn*V (11), *atp*H-*atp*I (10), *trn*K-*rps*16 (8), *trn*S-*trn*G (7), *rbc*L-*acc*D and *pet*A-*psb*J (6), and *ycf*3-*trn*S and *trn*L-*tr*nF (5). All SNPs showed variation in at least four groups. Tajima D values ranged from −1.80 to −1.59 and were found to be significant (*p* < 0.05) for *psa*A-*ycf*3, *rpl*32-*trn*L, *atp*H-*atp*I, *trn*S-*trn*G, and *trn*K-*rps*16. Neutral IGS spacers showed haplotype numbers (h) ranging from 1–6, and the maximum was recorded for *rbc*L-*acc*D, *rps*16-*trn*Q, and *trn*L-*trn*F. The IGS *rbc*L-*acc*D accumulated unique SNPs for CB, CCha, CAG, and CC and common SNPs for the CFG subclade. The IGS *rps*16-*trn*Q accumulated enough SNPs to differentiate all groups and species/variety except for the CC, CF, or CG species. After masking the *trn*F pseudogene^11,18^, *trn*L-*trn*F recorded unique SNPs for CB (2), CCha (1) and CAG (1) and a common SNP in the CA subclade (Fig. 3a).

Among the intronic regions, 36 SNPs were recorded from 13 introns. The number of SNPs ranged from 1–7 sites, and the maximum number of SNPs was found in the *ndh*A intron, which was the only intron that showed variation in at least four groups; however, the Tajima D value was found to be significant (D = −1.63, *p* < 0.05; Fig. 3a).

##### Coding DNA sequences and pseudogenes

Among coding DNA sequences, 131 SNPs were recorded from 45 CDSs, and the number of SNPs ranged between 1 and 28 sites. The maximum number of SNPs was found in the *ycf*1 CDS, followed by *acc*D, *ndh*F, *rpo*B, and *rpo*C2 (8 SNPs), all of which showed variation in more than three groups. In *ycf*1, 18 out of 28 SNPs were recorded in CB, followed by CCha (4), and six common SNPs were recorded within each complex and subcluster; however, none were specific to any of the A complex species/varieties. The *acc*D pseudogene accumulated SNPs for CB (3) and CCha (2), a unique SNP for CC and a common SNP among the A complex species/varieties. The SNPs in the *ndh*F CDS were recorded for CB (3), the CFG subclade (3), CG (2), and CAA (1). The *rpo*B CDS accumulated seven SNPs in CB, CCha, and the B complex, with a unique SNP for the CFG subclade. The *rpo*C2 CDS accumulated SNPs in CB (3), CCha (1), the B complex (2), the CAA/CAG subclade (1), and the A complex (1) (Fig. 3a).

The number of unique and common polymorphic sites was determined for each functional group including low polymorphic CDSs; the NADH oxidoreductase (ndh) CDS accumulated the highest number of unique and common SNPs (13) and enough to discriminate all species/varieties except the CC and CF subclades. The DNA-dependent RNA polymerase (rpo) and an unknown functional (ycf) CDSs accumulated 11 unique and common SNPs, with the highest number found in CB (3 SNPs each). The ycf group discriminated three species/varieties and four *Capsicum* groups, while the rpo group discriminated two species/varieties and four *Capsicum* groups. The small subunit of ribosome (rps), the large subunit of ribosome (rpl), and the subunits of ATP synthase (atp) CDSs accumulated 9, 8, and 7 unique and common SNPs, respectively. The other functional groups accumulated less than 5 SNPs, while the C-type cytochrome synthase gene (*ccs*A) was completely conserved (Fig. 3b).

The effect of SNPs on amino-acid changes was investigated by excluding the deletions in *acc*D, *rpl*20 and *ycf*1. Common and unique effects on each *Capsicum* group were determined and visualized in the form of interspecies/variety amino-acid-change frequency number. A total of 42 AA changes were detected: 15 were unique to a specific pan-plastome, and 17 changes were unique to one of the defined complexes or subclades. The highest number of unique AA changes was recorded from CB species, while CC, CF and CG showed no unique AA changes. Major AA changes were recorded toward an increase in isoleucine (I), one of which was exclusively found in the B complex species (leucine (L) to I), and valine (V) to I, which was exclusively recorded for the A complex species/varieties with the exception of a single record of a common AA change in the B complex. For the CFG group, alanine (A) was exclusively changed to threonine (T), glycine (G), and aspartate (D), and methionine (M) was changed to valine (V) (Fig. 3c).

##### rRNA and tRNA

The 16S rRNA showed a 10 bp variation at the 3′ terminus that was only recorded in one referenced CCha (GenBank: MH559328); however, this variation was not found in the other referenced CCha (GenBank: KX91318) or any of the sequenced accessions in the current study. Similarly, one SNP 109,254G > T was recorded uniquely in 23S rRNA for the referenced CAA (GenBank: JX270811). Both detected variations were conserved among the other accessions, including the CP species; thus, both were excluded from the current pan-plastome comparative analysis.

The tRNA alignments showed two intraspecific variations of unique changes in *trn*Q and *trn*G-GCC for a mislabeled CF accession and reference CG (GenBank: MH559322), respectively. The tRNA *trn*S-GGA, which is 87 bp in length, recorded a tree-variant SNP at position 21. The B complex was clearly separated from the A complex with a transition SNP (21/87 bp C > T), and CAG was separated from the other species/variety within the A complex with a transversion SNP (21/87 bp C > G). The SNP was found in the D-loop site and showed no effect on the secondary structure or the functionality of the *trn*S-GGA tRNA molecule (data not shown).

### Molecular marker development

#### *rpl*23-*trn*I length validation

**Fig. 4:**
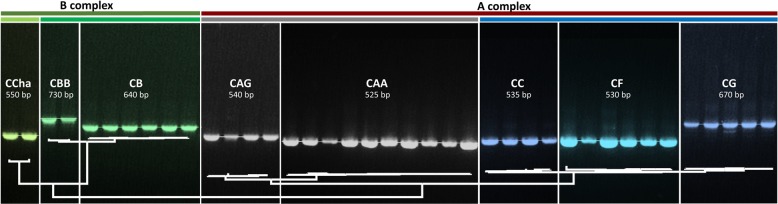
Colored and sized agarose gel electrophoresis shows *rpl*23-*trn*I loci amplified for representative non-gapped haplotypes of the studied *Capsicum* species/varieties. The amplified product was polymorphic and capable of discriminating the two complexes between 750–500 bp. Within the A complex (indicated by the red upper line), the product size showed a very narrow difference (~5–10 bp), except for CG, which shows a longer product of 670 bp; the CA species is indicated by a gray upper line, and the CFG clade is indicated by a blue upper line. Within the B complex (defined by dark-green underline), CCha (550 bp) is clearly smaller than CB (730–640 bp; defined with green underline) in product size. A clear difference was observed within the CB species: the highest in product size were CBB by label, while the low product sizes were of different varieties including some labeled as CBB. The product size differences reflect the same highly supported phylogenetic signal estimated by FastTree (bootstrap support >0.8) based on the variable sites of the whole plastome of the representative non-gapped haplotypes

Based on the variation in the loci size, *rpl*23-*trn*I was found to be variable in size mainly due to the 44 bp tandem repeat, which is suitable for downstream analysis. The IGS was tested for the capacity to discriminate the *Capsicum* cultivated species/varieties using a standard agarose gel electrophoresis protocol. *rpl*23-*trn*I was found to be highly polymorphic and capable of discriminating the two complexes (A and B), CFG clade, CB varieties, and CCha species (Fig. 4; Supplementary Fig. 5).

#### Phylogenetic signal

**Fig. 5:**
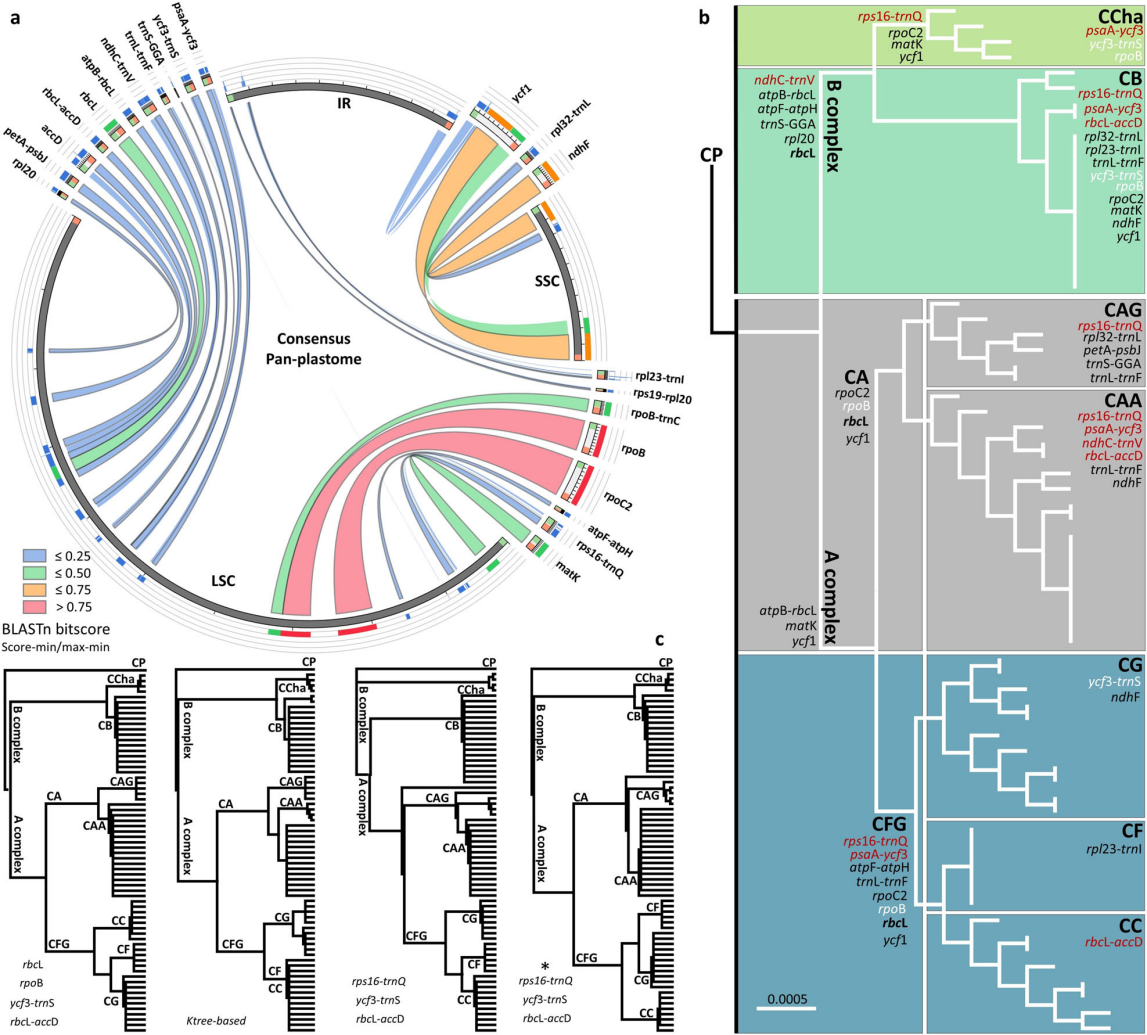
In silico validation of the hypervariable loci. **a** Hypervariability based on BLAST is shown in the Circos plot. The loci positions in the SSC, LSC and/or IR regions are shown; the line reflects the region size, and the color is based on the BLASTn score (less similar: <0.25 to the very similar: >0.75). **b** Maximum likelihood (ML)-based phylogenetic analysis for each hypervariable locus is indicated on the complete set tree shown in Fig. 2a based on clustering match. Loci with intraspecific variation are indicated in red, strong candidates from the current study are indicated in white, and strong candidates from the literature are indicated in bold. **c** The final ML trees produced from the tested combinations to retrieve the complete set of phylogenetic signals are shown. Trees from left to right are the visual-based tree, Ktree-based tree, loci with interspecific variation (InDels included) and the latter set when InDels are excluded (*)

A total of 22 hypervariable loci (including *mat*K, *atp*B-*rbc*L, and *rbc*L) were tested for their ability to retrieve the phyloplastomic signal, whether as a single locus or combined with others, using the maximum likelihood method. The hypervariability grade was confirmed by BLAST search of a consensus sequence for each locus to the consensus sequence of the pan-plastome (Fig. 5a). Sixteen loci were polymorphic due to SSRs, InDels and/or SNPs (BLASTn bitscore <0.5), and five loci were polymorphic due to SNPs only (BLASTn bitscore >0.5). Uniquely, *ycf*1 showed three levels of conservation, where the 5′ terminus was more variable than the 3′ terminus, and the intermediate area was more conserved; a similar result was found in *acc*D only when it was compared to the other members of Solanaceae^14^ but was not equal within the *Capsicum* plastomes.

When loci with intraspecific variation were not considered, two approaches were applied, one, visually, and two using the K-score of each tree constructed for each locus compared to the tree constructed based on the phyloplastome analysis. Based on the phylogenetic analysis of the haplotypes (see Fig. 2a), the loci of similar phylogenetic signals were indicated at each clade, subclade and mini-clade. As CB was considered the most diverse species, the major number of polymorphic loci (10) were capable of discriminating the species from the others. In contrast, CC and CF were uniquely discriminated from the other species/variety by *rbc*L-*acc*D and *rpl*23-*trn*I, respectively, followed by *ycf*3-*trn*S and *ndh*F to cluster CG and CB. Both *rpo*B and *rpo*C2, along with *rbc*L, were capable of defining the major clades (Fig. 5b). Therefore, a combined set based on the previously observed loci was tested. This set was capable of discriminating all the species/varieties; however, the CFG relationship was not correctly matched. Another combined set (Ktree-based) was tested using the k-score estimated for the top 8 loci, namely, *rbc*L, *mat*K, *rpo*B, *rpo*C2, *atp*B-*rbc*L, *atp*F-*atp*H, *trn*S, and *ycf*3-*trn*S. Similar to the visual-based set, CFG did not match correctly, with a lack of *rbc*L-*acc*D or *rpl*23-*trn*I, the CF and CC were clustered together. When loci with intraspecific variation were considered, a combined set of *ndh*C-*trn*V, *psa*A-*ycf*3, *rps*16-*trn*Q, and *rbc*L-*acc*D was tested. The strongest compatible phylogenetic signal was only found by *rps*16-*trn*Q, *rbc*L-*acc*D, and *ycf*3-*trn*S (added based on the visual inspection; Fig. 5c).

## Discussion

### Label corrections, taxonomic resolution, and incomplete lineage sorting

By comparing the five cultivated species (including the two distinguished varieties of CA) in terms of synteny and gene number, both were highly conserved and in agreement with the reference accession of CA (GenBank: JX270811). The GC% was 37.7% for all accessions, in accordance with previously reported values for the *Capsicum* plastomes^14–16,19–23^. Highly inconsistent GC% from different genera of the family Solanaceae have been reported; for example, species from the genus *Solanum* (*S. bulbocastanum*, *S. lycopersicum* and *S. tuberosum*) showed GC% = 37.9%, while the GC% was slightly different in the genus *Nicotiana*, where *N. tomentosiformis* and *N. tabacum* recorded GC% = 37.8 and 37.9%, respectively^14^. The plastome size expansion was previously reported as a distinct feature of the cultivated *Capsicum* (CA) when compared to other Solanaceae members^14^. One should expect that the plastome size differences would distinguish each species/variety. However, in the case in which each sample was assigned to a species/variety according to their herbarium labels, the plastome length showed high levels of size variation between and within each species/variety, which was in contrast to the anticipated results (Supplementary Fig. 1 and Supplementary Table 1).

In many herbaria, mislabeled or misclassified *Capsicum* collections have been reported, especially wild *Capsicum* species^9^. In the current study, 38 out of the 321 accessions were mislabeled, 21 of which were originally labeled as CAG but delimited as CG (14), CAA (4), and CF (3). The identified CG accessions were never morphologically identified as CG, either from the HUNAAS^17^ or USDA-ARS samples (Supplementary Table 1). Pereira-Dias et al.^24^ applied the genotyping-by-sequencing technique to investigate the population structure of cultivated *Capsicum* spp. collection (CAG, CAA, CB, CC, and CF) from the Spanish Center of Diversity. They detected two distinct genetic pools for the studied CAG accession, one of which clustered with CF and CC accessions in contrast to the other that was closely related to CAA, as taxonomically anticipated. A domesticated CAG form was hypothesized but could not explain the clustering with CF and CC; alternatively, the misclassification hypothesis was proposed. Based on the current phyloplastomic analysis supported by the referenced plastomes, the mislabeled CAG samples clustered near CC and CF were found to be CG accessions (Fig. 2; Supplementary Fig. 2). Hybridization between maternal CG and different parental species could be hypothesized to explain such incongruence between the herbaria label and the phylogenetic analysis due to the cross-pollination ability among the A complex species^7^. However, to obtain similar clustering patterns based on two approaches, pan-plastomes (based on maternally inherited markers) and GBS (nuclear-based markers) would strengthen the misclassification of CG accessions rather than the hybridization hypothesis.

On the other hand, the unexpected clustering of the referenced plastomes required a chronical review to investigate such incidences. The first complete pepper chloroplast genome was for the cultivated *C. annuum* L. (GenBank: JX270811)^14^. In 2015, the same plastome was resequenced for the Korean landrace “Subicho” (CAA; GenBank: KR078313)^15^ and the American bird pepper was reported (CAG; GenBank: KR078313)^19^. In 2016, the CAG plastome was resequenced (GenBank: KJ619462)^25^ and the complete plastome of *C. frutescence* (CF; GenBank: KR078312)^21^, the *C. baccatum* var. *baccatum* cp genome (CBB; GenBank: KR078314)^20^, and the complete sequence of *C. chinense* (CC; GenBank: KU041709)^22^ were reported. The phylogenetic analysis of the latter was found to be much closer to CAG, a wild progenitor of CA;^25^ due to the short time difference between both reports, CF was not yet included in the analysis. However, when a 129 bp longer genotype of *C. chinense* was resequenced one year later (GenBank: KX913217);^23^ the phylogenetic tree separated the two CC genotypes, while CF was included between the CA and CAA plastomes^22,23^. In 2018, an additional 11 genotypes were added to the pepper plastome data in GenBank, including three CAA, two CG, one CC and one CF from the A complex, one CCha, one CBB, one CBPe, and one *C. praetermissum* from the B complex, and a single genotype of CP from the P complex. Although the number of publicly available *Capsicum* cp genomes reached 23 plastomes (including CCha and CG, GenBank: KX913218 and KX913216, respectively; unpublished), the phyloplastomic analysis lacked all the deposited pepper plastomes; thus, the observed incongruence was never detected^11^. Based on the current analysis, among the deposited plastomes, *C. tovarii* (GenBank: KX913219)^16^, CF (GenBank: KR078312)^21^, and CAG (GenBank: KR078311)^19^, were found to be variants of CAA. The unexplained taxonomic position of one CA genotype and the CF plastomes (GenBank: MH559329 and MH559326) from D’Agostino et al.^11^ along with CAG (GenBank: KJ619462) were found to be part of the CG group. *Capsicum eximium* (GenBank: KX913220; unpublished) was found within the CF subclade (Fig. 2; Supplementary Table 5). Only with the application of the pan-plastome approach, the correct species assignment (at least maternally) of those accessions on the molecular level was possible.

Although taxonomists may be in a quandary as to whether species belonging to the A complex should be recognized as one or more taxonomical units (e.g., CC, CF, and CA were considered a single species^26^), within the commercial horticultural sector, five distinct species are recognized, among which CC, CF, and CAA are well distinguished. According to the collected material, the lowest number of mislabeled accessions was found when distinguishing CC and CF from CA (Supplementary Table 1; Supplementary Fig. 1). Molecular studies on *Capsicum* based on a chloroplast spacer (*atp*B‐*rbc*L) and a nuclear gene (*waxy*: gene encodes for an enzyme in granule-bound starch synthase pathway) showed that CA, CF, and CC are closely related; CC was relatively distant from CA, while CG was found between CF and CC^8^. In contrast, the only plastome-based analyses of cultivated *Capsicum* spp. found that CG was highly supported with two CA genotypes, leading to the conclusion that the CG taxonomic position was closer to CA than CC and CF; however, an unexplained genotype of CA was found to be highly related to CF and CC, forming a subclade within the A complex clade^11^.

In the current study, the inclusion of several plastomes of different species resolved a part of the unexplained incomplete lineage sorting (ILS) between marker-based and plastome-based phylogenetic analysis. In contrast to previously reported cytological evidence^9^, at the plastome level, the position of *C. chacoense* as a member of the B complex was confirmed^11^. CG was placed within the CFG subclade as a part of the A complex; however, CG was well separated from CC and CF (Fig. 2). CG is an endemic *Capsicum* species to the Galapagos Islands^5^; thus, the phylogenetic placement and subclustering of the CG group raises several key questions regarding (1) whether the identification of several mistakenly labeled CG accessions from several geographic regions challenges the endemism description of that species, (2) the identity of the mainland species that gave rise to it (a question that was previously proposed)^5^, and (3) whether a sister inland line was evolved in parallel to the island line.

### Pan-plastome and hypervariable regions

The pan-plastome constructed from multiple accessions per species/variety was expected to facilitate the detection of interspecific variation while avoiding the intraspecific variation that misled the development of non-precise and inapplicable molecular markers. The pan-plastome approach was previously applied to elucidate DNA barcodes for members of the family Zingiberaceae^27^ and for diversity assessment and phyloplastomics in the genus *Brachypodium*^28^. However, the success of the pan-plastome approach is based on the number of fully assembled accessions per species/variety, which in many cases can be very challenging.

The changes in the size of *acc*D and *rpl*20 were previously reported^14^, and *ycf*1, *rpl*32-*trn*L, and *trn*L-*trn*F were additionally described^11^. The repeats detected in *acc*D have been extensively reported in Solanaceae members^29^, even though the repeats cause no frameshifts or loss of function^14^. However, recently, the annotation of *acc*D has been considered an artifact^30^. Moreover, the *trn*L-*trn*F spacer was previously tested in *Capsicum* spp., where three InDels of 9, 16, and 71 bp were detected^31^. In the current study, when CP was excluded, four InDels of 6, 13, 14, and 19 bp were detected among all samples, while two more InDels were formed, with one of 6 bp in addition to the +98 bp InDel that was first described when CP was included^11^. The IGS *trn*L-*trn*F has previously recorded an important evolutionary event in Solanaceae, where a *trn*F duplicate (a pseudogene) was defined and its copy number was recorded for CB (4), CC (4), and CP (6)^18^. When testing whether the reported InDel^11^ was due to the *trn*F pseudogene copy^18^, the InDel showed two copies of the *trn*F pseudogene in addition to the original *trn*F gene. Notably, CP was previously reported to lack the original *trn*F gene^18^, which was not the present case. Two IGSs were previously proposed for *Capsicum* spp. identification; however, their discriminative capacity was limited to resolving either CP (*trn*L-*trn*F) or CB (*ccs*A-*ndh*D) from the other species/varieties^11^. In the current study, the variable tandem repeats located in *rpl*23-*trn*I were found to be highly polymorphic and capable of discriminating the studied *Capsicum* spp. and have never been reported. Three out of the five polymorphic SSRs were previously reported^11^, while the polymorphic SSRs located in *rps*16-*trn*Q and *ndh*C-*trn*V were never have been reported.

The highest AA changes were recorded toward isoleucine, and changes were observed from leucine to isoleucine (exclusively found in B complex species) and valine to isoleucine (mostly found in A complex species). Isoleucine has an important evolutionary aspect, sharing the first and second nucleotide of its codon (ATH, where H is T, C, or A) with methionine (ATG). The codon reassignment of ATA into ATG was hypothesized during the mitochondrial genome evolution in fungal species^32^, while it was recently reported during the chloroplast genome evolution of five *Chloroparvula* species (tiny green algae)^33^. Further analysis is required to study the impact of the observed AA changes, especially toward isoleucine, on *Capsicum* chloroplast evolution.

### Plastid molecular markers for cultivated *Capsicum* spp.

Exploring the single nucleotide polymorphisms uniquely recorded for each pan-plastome and shared between the defined groups facilitated the precise selection of hypervariable loci. A strong reliance on cpDNA loci in DNA barcoding and molecular systematics assumes that the gene tree of chloroplast sequences reflects the history and population dynamics of the species. However, non-neutrality in chloroplast genes may significantly affect the tree structure^34^. IGSs recorded ~3.8 vs. ~2.9 SNPs on average for CDS, while introns were third most abundant with ~2.7 SNPs on average. Coding loci can exhibit reduced levels of ILS compared to noncoding loci^35^. Such reduced ILS could be helpful in building complex phylogenies with rapid radiations^36^, but it will certainly distort estimated branch lengths when coalescent methods, which assume neutrality, are used. Selecting the candidate loci for validation as molecular markers was based on (a) variation in at least four groups (minimum major cluster number), (b) a haplotype number >4, and (c) neutrality when Tajima D was insignificant (*p* > 0.05), in addition to those loci that were previously reported by similar studies^11,31^. The selected candidates were tested for phylogenetic congruence with phyloplastomic signals separately and combined according to two approaches.

The intergenic spacers *trn*S-*trnf*M, *trn*T-*trn*L, *trn*H-*psb*A, *trn*L-*trn*F, *trn*D-*trn*T, *rpo*B-*trn*C, *rps*16, and *mat*K and the single-copy nuclear *waxy* introns have been previously tested on cultivated *Capsicum* spp., where *trn*T-*trn*L, *trn*L-*trn*F, and *trn*H-*psb*A could distinguish CA, CC, and CF^31^. In our study, none sufficiently reflected the phyloplastomic signal. The IGS *rps*16-*trn*Q was one of the most hypervariable loci detected in terms of polymorphism types. It showed a polymorphic SSR within and among the A complex species/varieties, accumulated the second highest number of SNPs (24 after *ycf*1, 28) and recorded the highest haplotype number (6). Similarly, the IGS *psa*A-*ycf*3 showed a polymorphic SSR event within and among the CA varieties and CG and accumulated the third highest number of SNPs (21). Unfortunately, these loci individually lack variations in closely related species (e.g., CFG species). Therefore, a concatenation of many individual loci was used to improve the resolution of the phylogenetic analysis. When validating *rps*16-*trn*Q and *psa*A-*ycf*3, both showed very similar phylogenetic signals. Both loci were tested with several additional candidates, *ycf*3-*trn*S and *rbc*L-*acc*D, which both accumulated enough SNPs to discriminate both CG and CC and consequently resolved CF within the less diverse CFG group. The combined IGS *rps*16-*trn*Q + *ycf*3-*trn*S + *rbc*L-*acc*D dataset has never been used for *Capsicum* and was capable of retrieving the phyloplastomic signal in two ways. First, when the gaps were included, the set was capable of resolving the CFG species correctly; however, the cultivated species/varieties appeared as one structured monophyletic group; in contrast, when the gaps were excluded, the tree restored the paraphyletic form but resorted the CFG species in the phyloplastome CFG order.

## Conclusion

Prior to pan-plastome construction, phyloplastomic and network analyses for 321 de novo assembled plastomes revealed different species assignations from their labels. The mislabeling incidences were found in both the studied samples and some of the NCBI-deposited plastomes. The reason for the delimitation of the *C. baccatum* varieties was not clear. Recombination detection and species delimitation analyses ensured species and/or variety clustering prior to pan-plastome construction for each studied species/variety. Gene synteny and number were highly conserved and showed a consistent GC% within *Capsicum* spp. compared to relative species. The plastomic features were compared and analyzed for pronounced neutral genetic variation to elucidate molecular markers and to understand the evolutionary dynamics affecting the cultivated species/varieties among the three *Capsicum* complexes (A, B, and P). Several evolutionary observations were recorded: (a) high genetic conservation at the rRNA and tRNA levels, (b) IGSs accumulated more genetic variation than the CDSs or the intronic regions, (c) the ndh gene complex accumulated the highest number of mutations, (d) unequal AA changes toward isoleucine were detected, where L to I was exclusively found in the B complex species, and V to I was mostly recorded in the A complex species/varieties, (e) large InDels, were present within *acc*D, *rpl*20 and *ycf*1, as previously reported, in addition to *rpl*23-*trn*I, *rps*16-*trn*Q, and *psa*A-*ycf*3, which have never been reported. The IGS *rpl*23-*trn*I was used as an electrophoresis-based molecular marker due to a 44 bp repeat unit that was validated to discriminate the studied species more efficiently than *trn*L-*trn*F (variation limited to CP) and *ccs*A-*ndh*D (variation limited to CB). The IGSs *rps*16-*trn*Q or *psa*A-*ycf*3 were found to be capable of retrieving the phyloplastomic signal when combined with *rbc*L-*acc*D and *ycf*3-*trn*S. Those loci are proposed as strong candidates for genetic diversity assessment and molecular identification among and within the cultivated *Capsicum* complexes. Although retrieving the whole plastome has become relatively inexpensive, the proposed single/multilocus molecular markers are recommended as a practical solution for germplasm banks to validate their pepper species ID database.

## Materials and methods

### Plant material

The species and lineages used in the current study were the same accessions sequenced in our previous study^17^ from the Institute of Vegetable Research, Hunan Academy of Agricultural Science, Changsha, China (HUNAAS); in addition to 65 samples newly retrieved and sequenced from the United States Department of Agriculture, Agriculture Research Services, USA (USDA-ARS). The samples represented the following cultivated *Capsicum* species/varieties: *C. chacoense* species “CCha”, *C. baccatum* species “CB” including two varieties (var. *baccatum* “CBB” and var. *pendulum* “CBPe”) representing the B complex; *C. annuum* species “CA” including two varieties (var*. annuum* “CAA” and var. *glabriusculum* “CAG”), *C. chinense* species “CC”, and *C. frutescens* species “CF” representing the A complex (Supplementary Table 1).

### DNA and library preparation

DNA extractions were performed using a DNeasy plant mini kit (Qiagen, Germantown, Maryland, USA). An Illumina paired-end DNA library was constructed using the Illumina TruSeq library preparation kit following the manufacturer’s instructions (Illumina, San Diego, California, USA). Paired-end whole-genome shotgun resequencing using Illumina HiSeq 4000 (NovoGene, Beijing, China) with ~300 bp insert size at 11× sequencing depth was performed.

### Single contig assembly

Since chloroplast genomes are abundant with several copies per cell, a 0.1–5% portion of the total reads per sample were de novo assembled using a Geneious assembler to reduce the exhaustive time and computational power needed to process 100% of the reads. Retrieving a single long contig (~130 kb) was the criterion for selecting the portion (%) of reads, as the % increased when the contig was not assembled, or the LSC-IR-SSC regions were partially incomplete from the termini. The clean pair-end data were mapped to the single long contig with 5 iteration times and checked for variants. The highest in quality was retained when variant and/or ambiguity sites were ≤2 sites per 150 bp window size. If the sites were >2 sites, the window was used as a reference to remap the clean reads of the accession with 15 iteration times. De novo assembly and mapping were performed using Geneious assembler and mapper implemented in Geneious R11^37^.

### Plastid gene annotation

The three main structural forms (LSC, SSC, and IR) were detected based on the repeating boundaries, annotated and separated for orientation correction and multiple comparisons, while the complete plastomes were joined and curated exhaustively. For each accession, single contigs were annotated as a circular molecule using GeSeq-Annotation of Organellar Genomes^38^. The HMMER profile search based on Embryophyta chloroplast (CDS and rRNA) was applied, while tRNAscan-SE V2^39^ was used to find and annotate tRNA genes. All reading frames of the protein-coding genes were verified by translation using Geneious R11.

### Alignment, phylogenetic inference, and species delimitation

All chloroplast genomes were aligned using MAFFT v7.164^40^, and free-gap ends were allowed. For the final assemblies of the chloroplast genomes, the IRb region was trimmed to the least that the *ycf*1 gene shared between the SSC and IRb regions and fully annotated to avoid repeated informative sites and decrease the computational process. In addition to the analyzed samples, *Capsicum* plastomes publicly available from the NCBI GenBank repository were included (Supplementary Table 5). Alignment gaps with a percentage of ≥99.6% among the aligned plastomes were excluded from further analysis.

The BI method was performed for phylogenomic analyses using the MrBayes 3.2.6^41^ plugin developed by Marc Suchard and Geneious team. The BI search was based on the HKY85 model, with two sets of four chains run for 1.1 million generations, while the subsampling frequency was every 200th generation. A 50% majority rule consensus tree was computed with a burn-in length of 100,000. The published plastome of *C. pubescens* (GenBank: MH559325^11^) was used as an outgroup. Clades were defined, and species were delimited using the species delimitation plugin^42^. Haplotype network analysis was conducted while excluding InDels using POPART^43^. FastTree^44^ was used to construct maximum likelihood (ML) phylogenetic trees using the generalized time‐reversible plus gamma distribution substitution model (GTR + G) and using the default settings. Trees were compared and scored using the Ktree-dist program^45^. The potential existence of interplastome recombination was assessed through the recombination detection methods in RDP4 v.4.56^46^ using default settings.

### Pan-plastome analysis

Because there were multiple accessions per species and a considerable amount of variation in different accessions from the same species/variety, a supra-plastome (a.k.a pan-chloroplast genome or pan-plastome) was constructed for each species/variety. Each group of accessions delimited as a species/variety was collapsed as one consensus sequence, considering 100% of differences (most ambiguous), while converting InDels into Ns. Seven pan-plastomes were generated and realigned. Sites with any ambiguity or Ns were masked; the final alignment was subjected to large gap, polymorphic SSR, and interpan-SNP (SNP) detection using Geneious R11. Tandem repeats were identified using Tandem Repeat Finder^47^. OGDRAW^48^ was used to produce a map of a consensus pan-plastome. The length in bp, number of mutations (Eta), number of haplotypes (H), haplotype diversity (Hd), and Tajima D neutrality test were calculated using DNASP V6^49^. Unique and common amino-acid changes among the studied pan-plastomes were demonstrated using Circos 0.69^50^.

### Primer design, PCR, and wet-lab validation

The selected loci were amplified by new primers designed using the primer design tool in Geneious R11 (Supplementary Table 6). PCRs were carried out using EasyTaq DNA Polymerase (Transgen Biotech, Beijing, China) following the manufacturer’s manual. A standard thermocycling profile was applied, and the annealing temperature was successful at 55 °C for all primers. Amplicons were revealed using EtBr-stained 1.5% agarose gel electrophoresis under UV light. For loci that accumulated high interspecies/variety SNPs, when successful, the PCR product was subjected to purification and sequencing (Tsingke, Wuhan, China).

## Supplementary information

Supplementary Figures and Tables

## Data Availability

The pan-plastome of each species/variety under study is deposited in the NCBI GenBank database under the following accession numbers: CB: *Capsicum baccatum* (CB; MN196481), *Capsicum chacoense* (CCha; MN196483); *Capsicum annuum* var. *annuum* (CAA; MN196479), *Capsicum annuum* var. *glabriusculum* (CAG; MN196480); *Capsicum chinense* (CC; MN196482), *Capsicum frutescens* (CF; MN196484), and *Capsicum galapagoense* (CG; MN196485).
