# Supplementary material for: Pan-plastome approach empowers the assessment of genetic variation in cultivated Capsicum species
Source: Hortic Res. 2019 Sep 7;6:108. doi: 10.1038/s41438-019-0191-x (PMC6804749; doi:10.1038/s41438-019-0191-x)
Supplement: Supplementary file 1 — Supplementary Figures and Tables [file 41438_2019_191_MOESM1_ESM.doc]

**Supplementary Material**

**Supplementary Figures**

| 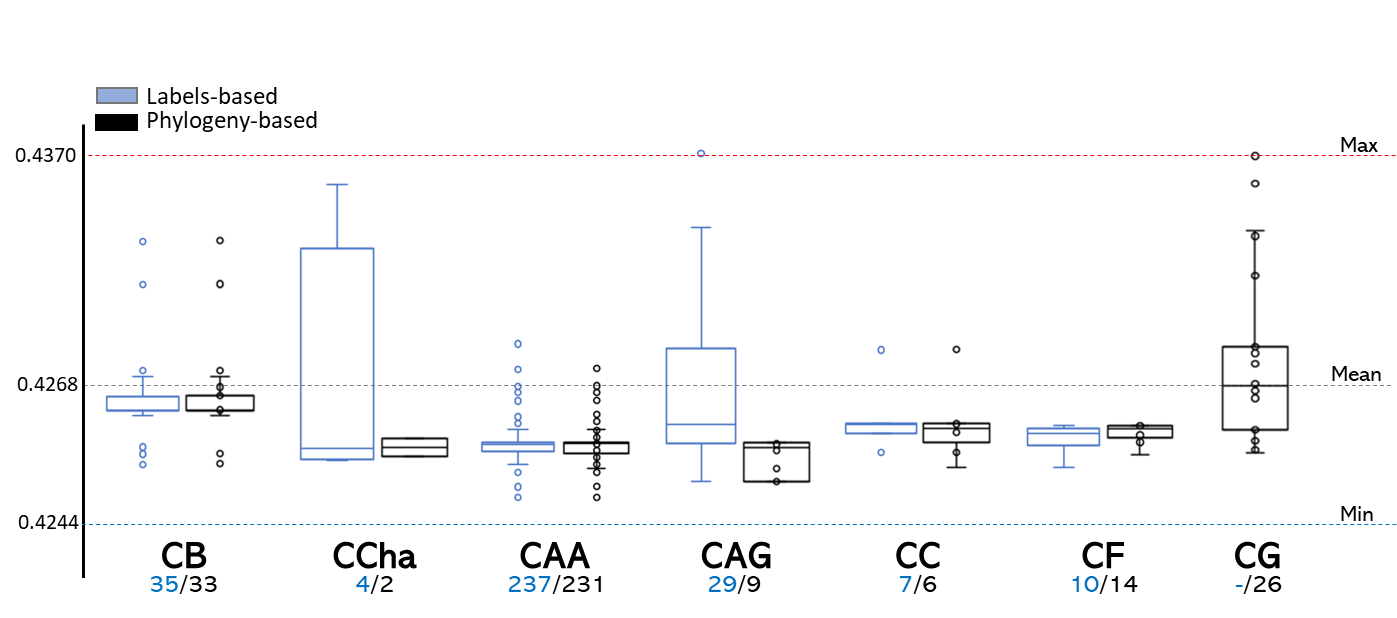 |
| --- |
| **Supplementary Figure 1.** Relative structural plastome size (rSPS) estimated based on herbaria labels (Blue) and Phylogenetic analysis (Black) for each sample as (LSC/IR)/(SSC/IR) length in bp. Mean, minimum and maximum relative structural plastome size are indicated regardless of the assigned groups. Based on the phylogenetic analysis, reduction in the rSPS and accession counts is observed in CAG, CCha and CF groups, increase is observed in CC group, while CAA and CB show similar variation with a slight reduction in accession counts of 7 and 9, respectively. None of the studied accessions was ever been labeled as CG. |

| 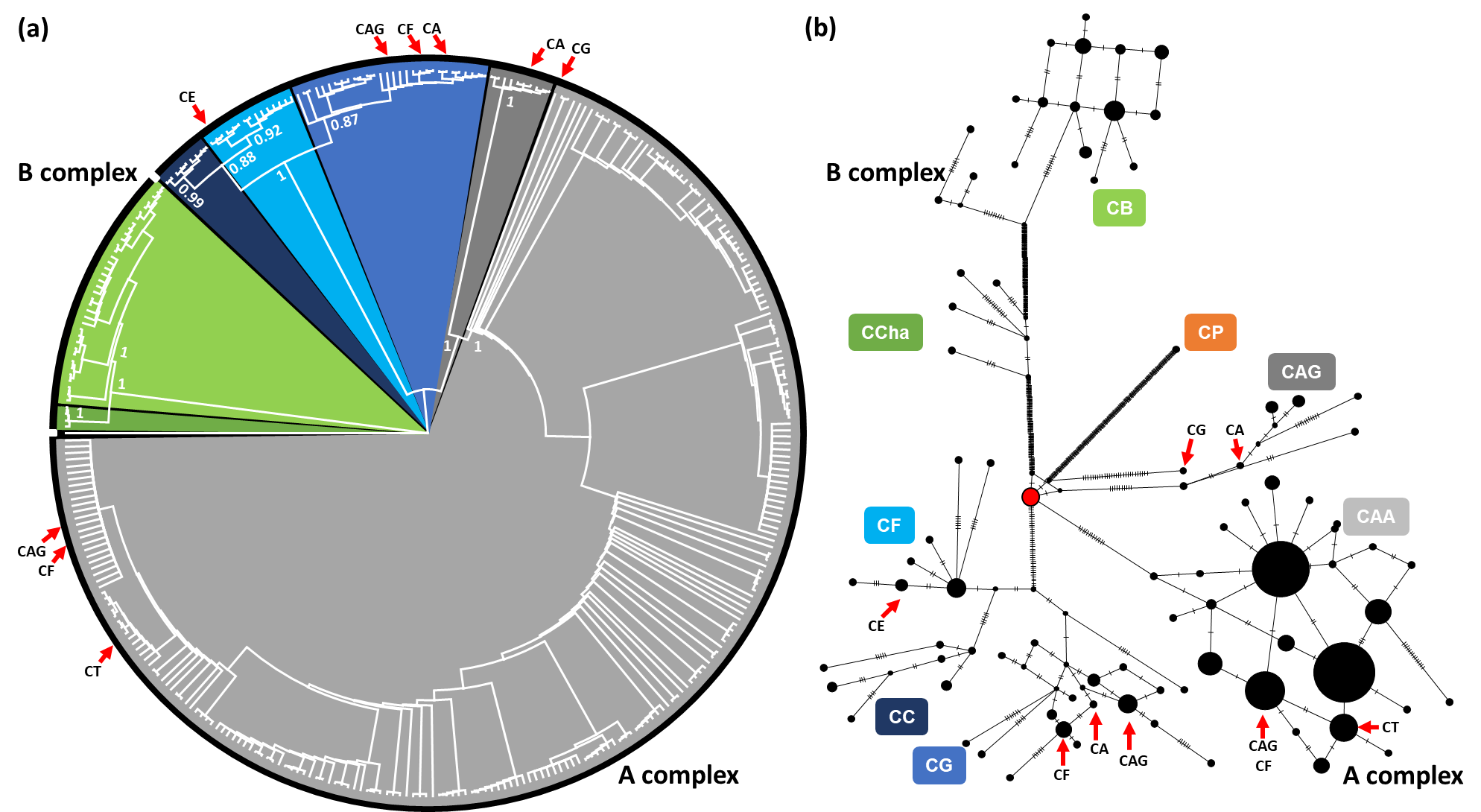 | **Supplementary Figure 2.** Phylogenomic analysis of the 212 gapped-haplotypes based on (a) BI methods and (b) Minimum spanning network. For both parts of the figure, CP was used as an outgroup/root; Each species shows a separate clade from other species. The A complex is formed by CAA, CAG, CC, CF and CG, where the phylogenetic signal is equal between the BI tree and the network for the CC, CF and CG species. The B complex is formed by CCha and CB, while its clearly resolved the position of the CCha in between the CB and the A complex species. Red node represents the most common ancestral connection among the groups. |
| --- | --- |

| 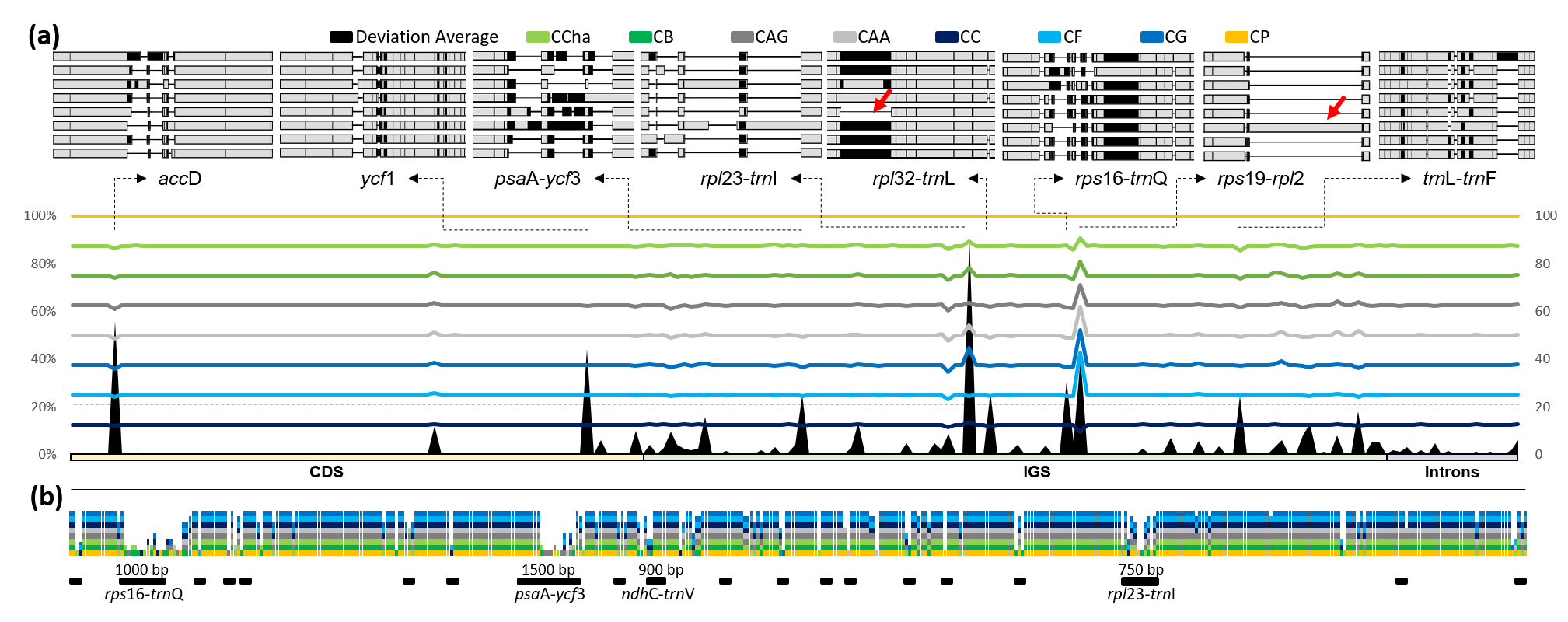 |
| --- |
| **Supplementary Figure 3.** Large InDels, SSR and Tandem Repeats plots. (a) Linear plot of loci sizes for each species/variety relative to the CP (top line) in percentages (left axis); deviation average score for each loci is shown (black peaks), where the minim deviation average >25 is marked by gray dotted lines (right axis). Alignmnet of the loci with high deviation average are shown; red arrows point to unique InDel event. (b) The repeats frequency for each pan-plastome were plotted as a stacked column plot, for both the detected SSR and tandem repeats; Regions with suitable sizes for downstream analysis were annotated by loci name and approximate size in bp. |

| 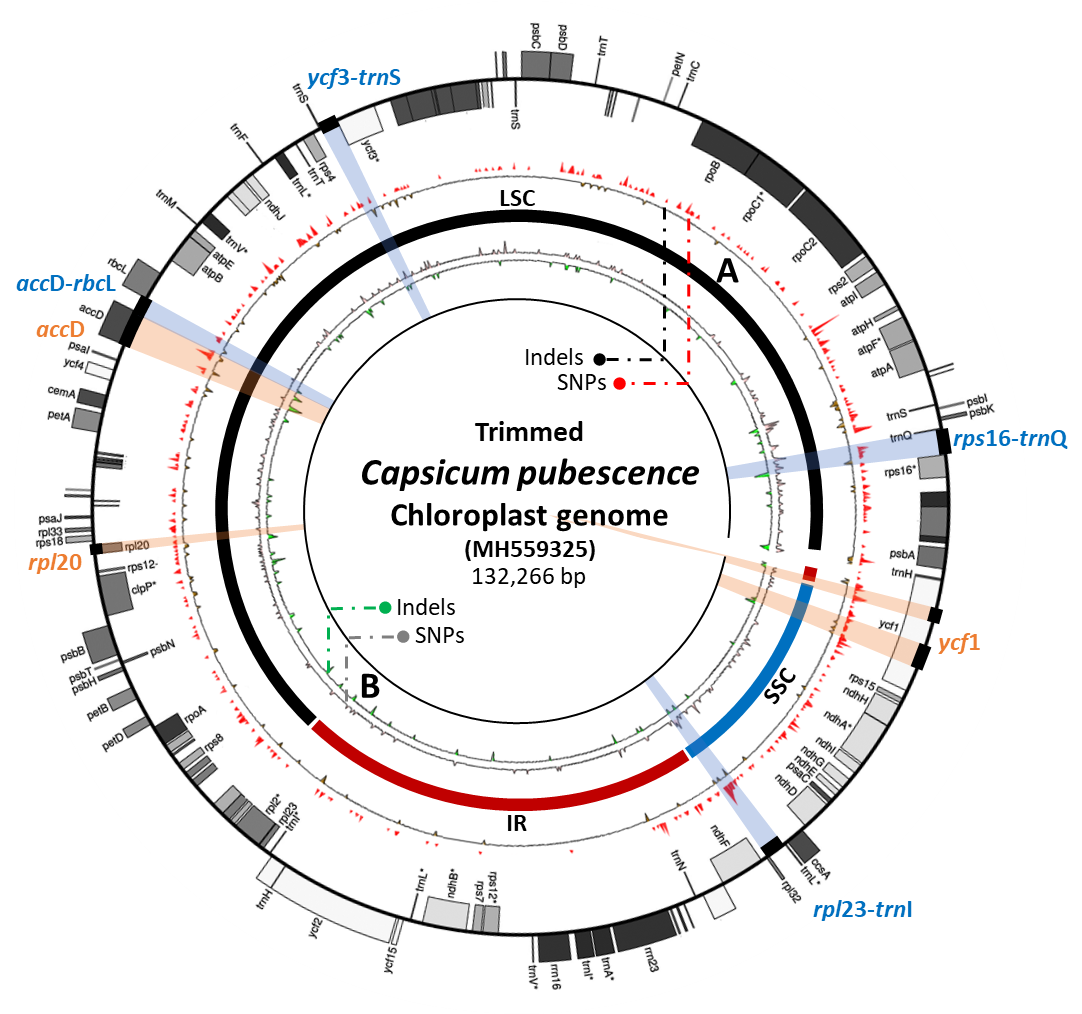 |
| --- |
| **Supplementary Figure 4.** SNPs based genetic variation among the cultivated *Capsicum* species/variety under study. The outer circle is the trimmed reference *Capsicum* *pubescens* plastome (details in Fig. 1). Two tracks A and B representing the inter and intra-species/variety genetic variation, respectively. For each track, the InDel-based sites (inwards) and SNP-based sites (outward) are drawn. It is obvious the low number of SNPs recorded within the IR regions, reflecting a major conservation process related to its structure, while the number of mutations is related to the single copy length, were the longer single copy accumulated more mutations, thus more common SNPs were more frequent than the short single copy. The CDS showed high genetic variation (InDels, repeats and SNPs) are highlighted in clear orange, while the IGS accumulated mutation capable to retrieve the Phylo-plastomic signal are highlighted in clear blue. |

| **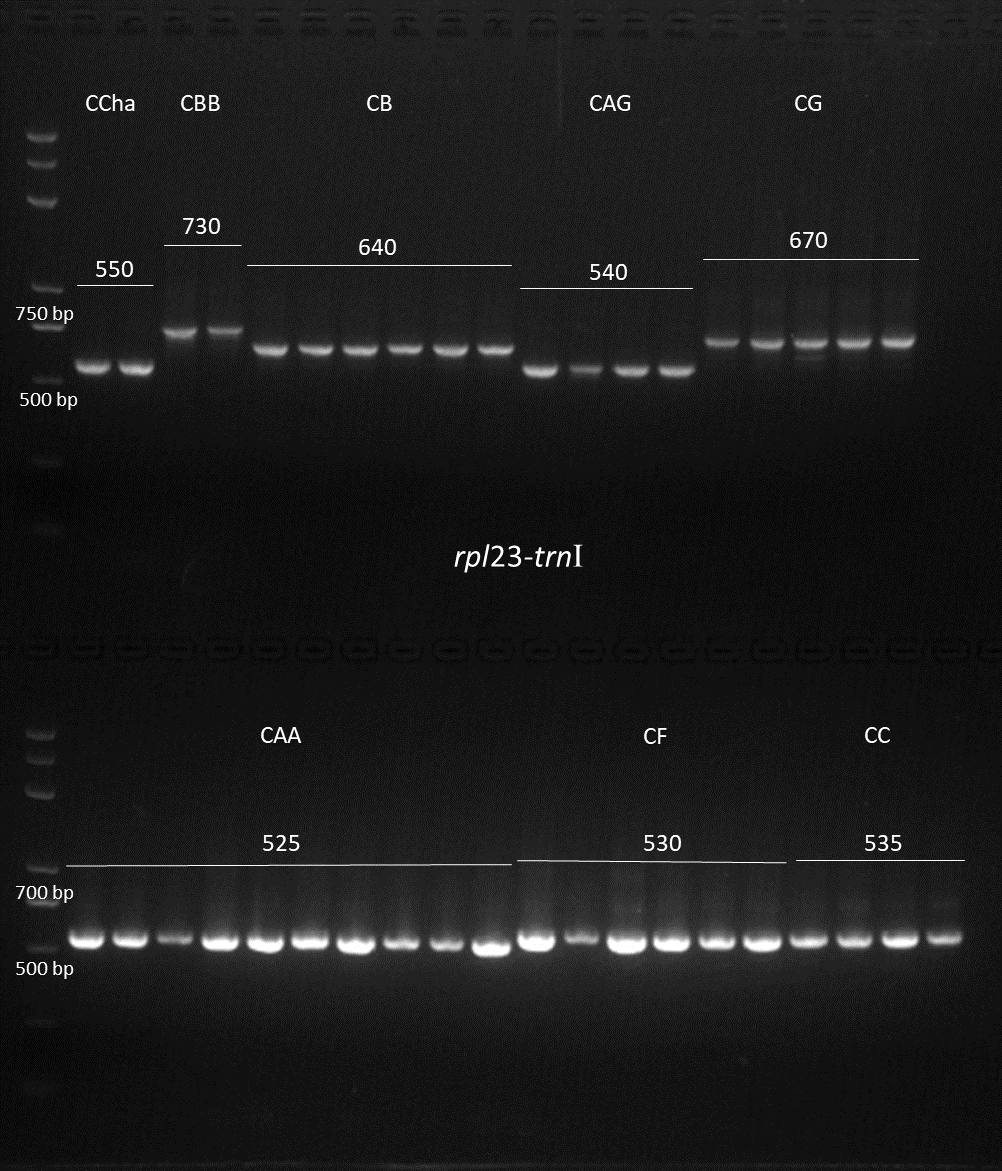** |
| --- |
| **Supplementary Figure 5.** Agarose gel electrophoresis shows *rpl*23-*trn*I amplified for representative samples of the *Capsicum* species/varieties under study. The amplified product was found polymorphic, and capable of discriminate the two complexes between 750-500 bp. Upper row, includes CCha (550 bp), CBB (730 bp), CB (includes different varieties; 640 bp) samples from the B complex along with CAG (540 bp) and CG (670 bp) samples from A complex. The second row includes CAA (525 bp), CF (530 bp) and CC (535 bp) samples to complete the A complex species/varieties under study. |

**Supplementary Tables**

**Supplementary Table 1.** The complete list of the successfully assembled plastomes. The sample source, identifier in the source database, name and country (the source’s country was written when the original source was not indicated). For each plastome, the total length (length), long single copy (LSC), short single copy (SSC), and inverted region (IR) lengths are recorded in bp. For each sample, the species assigned by the source (Label) and by the phylogenetic analysis (Phylogeny) are indicated.

| **Source** | **Identifier** | **Sample** | **Country** | **Length** | **LSC** | **SSC** | **IR** | **Label** | **Phylogeny** |
| --- | --- | --- | --- | --- | --- | --- | --- | --- | --- |
| **HUNAAS** | 16CS-01 | CS1 | China | 156733 | 87296 | 17853 | 25792 | CAA | CAA |
| **HUNAAS** | NX861 | CS11 | China | 156817 | 87380 | 17853 | 25792 | CAA | CAA |
| **HUNAAS** | NX1001 | CS12 | China | 156733 | 87296 | 17853 | 25792 | CAA | CAA |
| **HUNAAS** | A112 | CS13 | China | 156817 | 87380 | 17853 | 25792 | CAA | CAA |
| **HUNAAS** | TNX344 | CS14 | China | 156718 | 87395 | 17853 | 25735 | CAA | CAA |
| **HUNAAS** | NX805 | CS16 | China | 156819 | 87380 | 17853 | 25793 | CAA | CAA |
| **HUNAAS** | TNX348 | CS17 | China | 156703 | 87380 | 17853 | 25735 | CAA | CAA |
| **HUNAAS** | HNX192 | CS18 | China | 156806 | 87395 | 17853 | 25779 | CAA | CAA |
| **HUNAAS** | 16CS-02 | CS2 | China | 156817 | 87380 | 17853 | 25792 | CAA | CAA |
| **HUNAAS** | CJ12-17-1 | CS20 | China | 156958 | 87342 | 17912 | 25852 | CAA | CG |
| **HUNAAS** | S62 | CS23 | China | 156701 | 87290 | 17853 | 25779 | CAA | CAA |
| **HUNAAS** | A104 | CS24 | China | 156797 | 87358 | 17853 | 25793 | CAA | CAA |
| **HUNAAS** | A108 | CS25 | China | 156718 | 87395 | 17853 | 25735 | CAA | CAA |
| **HUNAAS** | NX718 | CS27 | China | 156836 | 87298 | 17860 | 25839 | CAA | CG |
| **HUNAAS** | NX79 | CS28 | China | 156958 | 87342 | 17912 | 25852 | CAA | CG |
| **HUNAAS** | CNX426 | CS29 | China | 156954 | 87338 | 17912 | 25852 | CAA | CF |
| **HUNAAS** | 16CS-03 | CS3 | China | 156725 | 87311 | 17846 | 25784 | CAA | CG |
| **HUNAAS** | NX968 | CS31 | China | 156729 | 87380 | 17853 | 25748 | CAA | CAA |
| **HUNAAS** | 16CS-04 | CS4 | China | 156791 | 87380 | 17853 | 25779 | CAA | CAA |
| **HUNAAS** | 16CS-05 | CS5 | China | 157046 | 87342 | 17912 | 25896 | CAA | CG |
| **HUNAAS** | 07H14-1 | CS7 | China | 157134 | 87342 | 17912 | 25940 | CAA | CG |
| **HUNAAS** | 07g5-2-4-1 | CS8 | China | 156805 | 87368 | 17853 | 25792 | CAA | CAA |
| **HUNAAS** | 1145-1 | CT10 | China | 156805 | 87368 | 17853 | 25792 | CAA | CAA |
| **HUNAAS** | 07HN28-1 | CT11 | China | 156805 | 87368 | 17853 | 25792 | CAA | CAA |
| **HUNAAS** | 06g38-3-1-1-1 | CT13 | China | 156821 | 87384 | 17853 | 25792 | CAA | CAA |
| **HUNAAS** | J04-46-2 | CT14 | China | 156817 | 87380 | 17853 | 25792 | CAA | CAA |
| **HUNAAS** | A113-1 | CT17 | China | 156947 | 87384 | 17853 | 25855 | CAA | CAA |
| **HUNAAS** | S12 | CT19 | China | 156817 | 87380 | 17853 | 25792 | CAA | CAA |
| **HUNAAS** | CJ10-26-3-1 | CT2 | China | 156857 | 87420 | 17853 | 25792 | CAA | CAA |
| **HUNAAS** | S37 | CT21 | China | 156817 | 87380 | 17853 | 25792 | CAA | CAA |
| **HUNAAS** | S42 | CT22 | China | 156831 | 87394 | 17853 | 25792 | CAA | CAA |
| **HUNAAS** | SJ06-41-1-1 | CT26 | China | 156831 | 87394 | 17853 | 25792 | CAA | CAA |
| **HUNAAS** | SJ09-72-1 | CT29 | China | 156805 | 87368 | 17853 | 25792 | CAA | CAA |
| **HUNAAS** | CNX85-1 | CT3 | China | 156819 | 87380 | 17853 | 25793 | CAA | CAA |
| **HUNAAS** | SJ07-39-2-1-1 | CT30 | China | 156817 | 87380 | 17853 | 25792 | CAA | CAA |
| **HUNAAS** | SJ08-55-1 | CT31 | China | 156781 | 87368 | 17853 | 25780 | CAA | CAA |
| **HUNAAS** | C51 | CT32 | China | 156731 | 87295 | 17852 | 25792 | CAA | CAA |
| **HUNAAS** | CJ10-32-1-1-1-1 | CT37 | China | 156921 | 87380 | 17853 | 25844 | CAA | CAA |
| **HUNAAS** | SJ08-51-1 | CT39 | China | 156832 | 87395 | 17853 | 25792 | CAA | CAA |
| **HUNAAS** | CJ11-1-1-1 | CT4 | China | 156805 | 87368 | 17853 | 25792 | CAA | CAA |
| **HUNAAS** | CNX329-1 | CT6 | China | 156943 | 87380 | 17853 | 25855 | CAA | CAA |
| **HUNAAS** | 0643-1 | CT8 | China | 156817 | 87380 | 17853 | 25792 | CAA | CAA |
| **HUNAAS** | CXJ-B33 | CXJ21 | China | 156817 | 87380 | 17853 | 25792 | CAA | CAA |
| **HUNAAS** | CXJ-B35 | CXJ22 | China | 156817 | 87380 | 17853 | 25792 | CAA | CAA |
| **HUNAAS** | CXJ-B0101 | CXJ23 | China | 156817 | 87380 | 17853 | 25792 | CAA | CAA |
| **HUNAAS** | CXJ-B129 | CXJ24 | China | 156688 | 87277 | 17853 | 25779 | CAA | CAA |
| **HUNAAS** | CXJ-B169 | CXJ25 | China | 156793 | 87382 | 17853 | 25779 | CAA | CAA |
| **HUNAAS** | CXJ-B193 | CXJ26 | China | 156801 | 87364 | 17853 | 25792 | CAA | CAA |
| **HUNAAS** | CXJ-B272 | CXJ28 | China | 156807 | 87370 | 17853 | 25792 | CAA | CAA |
| **HUNAAS** | CXJ-B276 | CXJ29 | China | 156791 | 87380 | 17853 | 25779 | CAA | CAA |
| **HUNAAS** | CXJ-B0511 | CXJ30 | China | 156791 | 87380 | 17853 | 25779 | CAA | CAA |
| **HUNAAS** | CXJ-A02 | CXJ37 | China | 156817 | 87380 | 17853 | 25792 | CAA | CAA |
| **HUNAAS** | CXJ-A37 | CXJ51 | China | 156819 | 87380 | 17853 | 25793 | CAA | CAA |

**Supplementary Table 1.** Continue.

| **Source** | **Identifier** | **Sample** | **Country** | **Length** | **LSC** | **SSC** | **IR** | **Label** | **Phylogeny** |
| --- | --- | --- | --- | --- | --- | --- | --- | --- | --- |
| **HUNAAS** | CXJ-A038 | CXJ52 | China | 156817 | 87380 | 17853 | 25792 | CAA | CAA |
| **HUNAAS** | CXJ-A039 | CXJ53 | China | 156735 | 87324 | 17853 | 25779 | CAA | CAA |
| **HUNAAS** | CXJ-A42 | CXJ55 | China | 156817 | 87380 | 17853 | 25792 | CAA | CAA |
| **HUNAAS** | CXJ-A48 | CXJ58 | China | 156887 | 87394 | 17853 | 25820 | CAA | CAA |
| **HUNAAS** | CXJ-A49 | CXJ59 | China | 156817 | 87380 | 17853 | 25792 | CAA | CAA |
| **HUNAAS** | CXJ-A52 | CXJ60 | China | 156832 | 87395 | 17853 | 25792 | CAA | CAA |
| **HUNAAS** | CXJ-A55 | CXJ61 | China | 156951 | 87380 | 17853 | 25859 | CAA | CAA |
| **HUNAAS** | CXJ-A71 | CXJ64 | China | 156819 | 87380 | 17853 | 25793 | CAA | CAA |
| **HUNAAS** | CXJ-A72 | CXJ65 | China | 156831 | 87394 | 17853 | 25792 | CAA | CAA |
| **HUNAAS** | CXJ-A76 | CXJ66 | China | 156791 | 87380 | 17853 | 25779 | CAA | CAA |
| **HUNAAS** | CXJ-A88 | CXJ67 | China | 156817 | 87380 | 17853 | 25792 | CAA | CAA |
| **HUNAAS** | CXJ-A096 | CXJ68 | China | 156835 | 87398 | 17853 | 25792 | CAA | CAA |
| **HUNAAS** | CXJ-A98 | CXJ69 | China | 156791 | 87380 | 17853 | 25779 | CAA | CAA |
| **HUNAAS** | CXJ-A100 | CXJ70 | China | 156807 | 87370 | 17853 | 25792 | CAA | CAA |
| **HUNAAS** | CXJ-A111-1 | CXJ72 | China | 156703 | 87380 | 17853 | 25735 | CAA | CAA |
| **HUNAAS** | CXJ-A114 | CXJ73 | China | 156729 | 87380 | 17853 | 25748 | CAA | CAA |
| **HUNAAS** | CXJ-A122 | CXJ76 | China | 156717 | 87394 | 17853 | 25735 | CAA | CAA |
| **HUNAAS** | CXJ-A125 | CXJ77 | China | 156791 | 87380 | 17853 | 25779 | CAA | CAA |
| **HUNAAS** | CXJ-A128-1 | CXJ79 | China | 156743 | 87339 | 17846 | 25779 | CAA | CG |
| **HUNAAS** | CXJ-A130 | CXJ80 | China | 156817 | 87380 | 17853 | 25792 | CAA | CAA |
| **HUNAAS** | CXJ-A131 | CXJ81 | China | 156945 | 87380 | 17853 | 25856 | CAA | CAA |
| **HUNAAS** | CXJ-A133 | CXJ82 | China | 156817 | 87380 | 17853 | 25792 | CAA | CAA |
| **HUNAAS** | CXJ-A143 | CXJ86 | China | 156819 | 87380 | 17853 | 25793 | CAA | CAA |
| **HUNAAS** | CXJ-A145 | CXJ87 | China | 156791 | 87380 | 17853 | 25779 | CAA | CAA |
| **HUNAAS** | CXJ-A148 | CXJ89 | China | 156817 | 87380 | 17853 | 25792 | CAA | CAA |
| **HUNAAS** | CXJ-A162 | CXJ92 | China | 156817 | 87380 | 17853 | 25792 | CAA | CAA |
| **HUNAAS** | CXJ-A163 | CXJ93 | China | 156910 | 87395 | 17853 | 25831 | CAA | CAA |
| **HUNAAS** | CXJ-A178 | CXJ99 | China | 156817 | 87380 | 17853 | 25792 | CAA | CAA |
| **HUNAAS** | CP06CA106 | CZM11 | Mexico | 156921 | 87368 | 17853 | 25850 | CAA | CAA |
| **HUNAAS** | CP06CA63 | CZM12 | Mexico | 156818 | 87407 | 17853 | 25779 | CAA | CAA |
| **HUNAAS** | CA06CA101 | CZM13 | Mexico | 156817 | 87380 | 17853 | 25792 | CAA | CAA |
| **HUNAAS** | CA06CA98 | CZM14 | Mexico | 156859 | 87346 | 17911 | 25801 | CAA | CAG |
| **HUNAAS** | CA06CA67 | CZM15 | Mexico | 156817 | 87380 | 17853 | 25792 | CAA | CAA |
| **HUNAAS** | CA06CA65 | CZM16 | Mexico | 156817 | 87380 | 17853 | 25792 | CAA | CAA |
| **HUNAAS** | CA06CA64 | CZM17 | Mexico | 156806 | 87395 | 17853 | 25779 | CAA | CAA |
| **HUNAAS** | HJ10-1 | HL14 | China | 156914 | 87381 | 17853 | 25840 | CAA | CAA |
| **HUNAAS** | HJ2011-1-1-1-1 | HL15 | China | 156819 | 87380 | 17853 | 25793 | CAA | CAA |
| **HUNAAS** | HNX159 | HL16 | China | 156703 | 87290 | 17853 | 25780 | CAA | CAA |
| **HUNAAS** | J03-111 | HL17 | China | 157042 | 87338 | 17912 | 25896 | CAA | CG |
| **HUNAAS** | HNX91 | HL19 | China | 156819 | 87380 | 17853 | 25793 | CAA | CAA |
| **HUNAAS** | CJ12-10-1-2 | HL2 | China | 156807 | 87368 | 17853 | 25793 | CAA | CAA |
| **HUNAAS** | LIU-16 | HP10 | China | 156791 | 87380 | 17853 | 25779 | CAA | CAA |
| **HUNAAS** | SJ08-1-1 | HP17 | China | 156831 | 87394 | 17853 | 25792 | CAA | CAA |
| **HUNAAS** | H1322 | HP8 | China | 156717 | 87394 | 17853 | 25735 | CAA | CAA |
| **HUNAAS** | LIU-50 | HP9 | China | 156805 | 87368 | 17853 | 25792 | CAA | CAA |
| **HUNAAS** | D70-3-1-1 | NJ10 | China | 156803 | 87392 | 17853 | 25779 | CAA | CAA |
| **HUNAAS** | SJ10-57 | NJ11 | China | 156817 | 87380 | 17853 | 25792 | CAA | CAA |
| **HUNAAS** | X7 | NJ13 | China | 156885 | 87380 | 17853 | 25826 | CAA | CAA |
| **HUNAAS** | 0 | NJ14 | China | 156807 | 87370 | 17853 | 25792 | CAA | CAA |
| **HUNAAS** | 03-71-1-1-1 | NJ15 | China | 156817 | 87380 | 17853 | 25792 | CAA | CAA |
| **HUNAAS** | 0601-2-6-1-3-2 | NJ16 | China | 156953 | 87380 | 17853 | 25860 | CAA | CAA |
| **HUNAAS** | 06HN12-1-1 | NJ17 | China | 156819 | 87380 | 17853 | 25793 | CAA | CAA |
| **HUNAAS** | L2014-40 | NJ21 | China | 156877 | 87380 | 17853 | 25822 | CAA | CAA |
| **HUNAAS** | C2 | NJ23 | China | 156953 | 87380 | 17853 | 25860 | CAA | CAA |
| **HUNAAS** | J01-7 | NJ25 | China | 156817 | 87380 | 17853 | 25792 | CAA | CAA |
| **HUNAAS** | SJ09-37-1-1 | NJ26 | China | 156817 | 87380 | 17853 | 25792 | CAA | CAA |
| **HUNAAS** | 16NJ-28 | NJ28 | China | 156869 | 87380 | 17853 | 25818 | CAA | CAA |
| **HUNAAS** | S58 | NJ29 | China | 156867 | 87430 | 17853 | 25792 | CAA | CAA |
| **HUNAAS** | SJ10-35-1 | NJ30 | China | 156867 | 87380 | 17853 | 25817 | CAA | CAA |
| **HUNAAS** | SJ08-58-1 | NJ31 | China | 156791 | 87380 | 17853 | 25779 | CAA | CAA |
| **HUNAAS** | L2014-78 | NJ33 | China | 156817 | 87380 | 17853 | 25792 | CAA | CAA |
| **HUNAAS** | SJ09-47 | NJ34 | China | 156703 | 87380 | 17853 | 25735 | CAA | CAA |

**Supplementary Table 1.** Continue.

| **Source** | **Identifier** | **Sample** | **Country** | **Length** | **LSC** | **SSC** | **IR** | **Label** | **Phylogeny** |
| --- | --- | --- | --- | --- | --- | --- | --- | --- | --- |
| **HUNAAS** | SJ05-71-2-1-1-1 | NJ35 | China | 156817 | 87380 | 17853 | 25792 | CAA | CAA |
| **HUNAAS** | SJ10-41 | NJ36 | China | 156807 | 87370 | 17853 | 25792 | CAA | CAA |
| **HUNAAS** | T11-9 | NJ37 | China | 156817 | 87380 | 17853 | 25792 | CAA | CAA |
| **HUNAAS** | SJ10-43 | NJ38 | China | 156817 | 87380 | 17853 | 25792 | CAA | CAA |
| **HUNAAS** | C99 | NJ39 | China | 156809 | 87372 | 17853 | 25792 | CAA | CAA |
| **HUNAAS** | C83 | NJ40 | China | 156834 | 87395 | 17853 | 25793 | CAA | CAA |
| **HUNAAS** | W23F2-4-2-1 | NJ44 | China | 156805 | 87368 | 17853 | 25792 | CAA | CAA |
| **HUNAAS** | SJ14-1 | NJ45 | China | 156819 | 87380 | 17853 | 25793 | CAA | CAA |
| **HUNAAS** | LIU-44 | NJ46 | China | 156819 | 87380 | 17853 | 25793 | CAA | CAA |
| **HUNAAS** | G13F2-3-1-1 | NJ47 | China | 156817 | 87380 | 17853 | 25792 | CAA | CAA |
| **HUNAAS** | NX412 | NJ48 | China | 156833 | 87394 | 17853 | 25793 | CAA | CAA |
| **HUNAAS** | 26-1-5-3-1 | NJ49 | China | 156817 | 87380 | 17853 | 25792 | CAA | CAA |
| **HUNAAS** | SJ07-116-2-1-1 | NJ50 | China | 156817 | 87380 | 17853 | 25792 | CAA | CAA |
| **HUNAAS** | PI 222975 01 SD | OLJ10 | Iran | 156769 | 87374 | 17853 | 25771 | CAA | CAA |
| **HUNAAS** | PI 297456 01 SD | OLJ14 | Spain | 156850 | 87441 | 17853 | 25778 | CAA | CAA |
| **HUNAAS** | PI 379119 01 SD | OLJ5 | Former Serbia and Montenegro | 156933 | 87380 | 17853 | 25850 | CAA | CAA |
| **HUNAAS** | A28 | QT10 | China | 156817 | 87380 | 17853 | 25792 | CAA | CAA |
| **HUNAAS** | A125 | QT14 | China | 156839 | 87380 | 17853 | 25803 | CAA | CAA |
| **HUNAAS** | S92 | QT16 | China | 156793 | 87382 | 17853 | 25779 | CAA | CAA |
| **HUNAAS** | A97、TC6903 | QT17 | China | 156818 | 87381 | 17853 | 25792 | CAA | CAA |
| **HUNAAS** | A120、VI013510 | QT18 | China | 156778 | 87341 | 17853 | 25792 | CAA | CAA |
| **HUNAAS** | perennial | QT2 | China | 156771 | 87341 | 17846 | 25792 | CAA | CG |
| **HUNAAS** | A124 | QT21 | China | 156753 | 87349 | 17846 | 25779 | CAA | CG |
| **HUNAAS** | A34 | QT8 | China | 156821 | 87384 | 17853 | 25792 | CAA | CAA |
| **HUNAAS** | 7801 | TJ02 | China | 156718 | 87395 | 17853 | 25735 | CAA | CAA |
| **HUNAAS** | T06-6 | TJ04 | China | 156746 | 87395 | 17853 | 25749 | CAA | CAA |
| **HUNAAS** | 9203 | TJ05 | China | 156703 | 87380 | 17853 | 25735 | CAA | CAA |
| **HUNAAS** | TNX241 | TJ06 | China | 156731 | 87380 | 17853 | 25749 | CAA | CAA |
| **HUNAAS** | T02-12 | TJ07 | China | 156819 | 87380 | 17853 | 25793 | CAA | CAA |
| **HUNAAS** | 16TJ-08 | TJ08 | China | 156745 | 87394 | 17853 | 25749 | CAA | CAA |
| **HUNAAS** | A3 | TJ09 | China | 156817 | 87380 | 17853 | 25792 | CAA | CAA |
| **HUNAAS** | 5905 | TJ1 | China | 156839 | 87402 | 17853 | 25792 | CAA | CAA |
| **HUNAAS** | T09-2 | TJ12 | China | 156703 | 87380 | 17853 | 25735 | CAA | CAA |
| **HUNAAS** | T05-16 | TJ13 | China | 156732 | 87380 | 17854 | 25749 | CAA | CAA |
| **HUNAAS** | TH09-11 | TJ14 | China | 156849 | 87380 | 17853 | 25808 | CAA | CAA |
| **HUNAAS** | T09-1 | TJ16 | China | 156817 | 87380 | 17853 | 25792 | CAA | CAA |
| **HUNAAS** | TNX403 | TJ17 | China | 156763 | 87380 | 17853 | 25765 | CAA | CAA |
| **HUNAAS** | TNX58 | TJ18 | China | 156831 | 87394 | 17853 | 25792 | CAA | CAA |
| **HUNAAS** | TNX96 | TJ19 | China | 156817 | 87380 | 17853 | 25792 | CAA | CAA |
| **HUNAAS** | 10L37F-1 | TJ21 | China | 156832 | 87395 | 17853 | 25792 | CAA | CAA |
| **HUNAAS** | TNX413 | TJ25 | China | 156931 | 87380 | 17853 | 25849 | CAA | CAA |
| **HUNAAS** | TNX300 | TJ26 | China | 156817 | 87380 | 17853 | 25792 | CAA | CAA |
| **HUNAAS** | T10-8 | TJ27 | China | 156819 | 87382 | 17853 | 25792 | CAA | CAA |
| **HUNAAS** | A58 | TJ28 | China | 156708 | 87383 | 17853 | 25736 | CAA | CAA |
| **HUNAAS** | TNX379 | TJ29 | China | 156844 | 87407 | 17853 | 25792 | CAA | CAA |
| **HUNAAS** | TH05-21-1-2-2-1 | TJ30 | China | 156805 | 87368 | 17853 | 25792 | CAA | CAA |
| **HUNAAS** | LIU-58 | TJ31 | China | 156799 | 87394 | 17853 | 25776 | CAA | CAA |
| **HUNAAS** | T13-3 | TJ32 | China | 156805 | 87368 | 17853 | 25792 | CAA | CAA |
| **HUNAAS** | A130 | TJ33 | China | 156817 | 87380 | 17853 | 25792 | CAA | CAA |
| **HUNAAS** | THNX343 | TJ34 | China | 156831 | 87394 | 17853 | 25792 | CAA | CAA |
| **HUNAAS** | TNX394 | TJ36 | China | 156753 | 87404 | 17853 | 25748 | CAA | CAA |
| **HUNAAS** | TNX397 | TJ37 | China | 156817 | 87380 | 17853 | 25792 | CAA | CAA |
| **HUNAAS** | TNX319 | TJ38 | China | 156729 | 87380 | 17853 | 25748 | CAA | CAA |
| **HUNAAS** | TNX296 | TJ39 | China | 156703 | 87380 | 17853 | 25735 | CAA | CAA |
| **HUNAAS** | TNX283 | TJ40 | China | 156833 | 87394 | 17853 | 25793 | CAA | CAA |
| **HUNAAS** | TNX103 | TJ41 | China | 156703 | 87380 | 17853 | 25735 | CAA | CAA |
| **HUNAAS** | TNX108 | TJ42 | China | 157428 | 87901 | 17853 | 25837 | CAA | CAA |
| **HUNAAS** | TNX106 | TJ43 | China | 156718 | 87395 | 17853 | 25735 | CAA | CAA |
| **HUNAAS** | TNX359 | TJ44 | China | 156817 | 87380 | 17853 | 25792 | CAA | CAA |
| **HUNAAS** | A62 | TJ46 | China | 156719 | 87396 | 17853 | 25735 | CAA | CAA |
| **HUNAAS** | T11-3 | TJ48 | China | 156718 | 87395 | 17853 | 25735 | CAA | CAA |

**Supplementary Table 1.** Continue.

| **Source** | **Identifier** | **Sample** | **Country** | **Length** | **LSC** | **SSC** | **IR** | **Label** | **Phylogeny** |
| --- | --- | --- | --- | --- | --- | --- | --- | --- | --- |
| **HUNAAS** | THNX83 | TJ50 | China | 156793 | 87380 | 17853 | 25780 | CAA | CAA |
| **HUNAAS** | T09-3-1 | TJ54 | China | 156817 | 87380 | 17853 | 25792 | CAA | CAA |
| **HUNAAS** | 9701 | XJ02 | China | 156809 | 87370 | 17853 | 25793 | CAA | CAA |
| **HUNAAS** | SJ05-12 | XJ04 | China | 156768 | 87357 | 17853 | 25779 | CAA | CAA |
| **HUNAAS** | SJ05-14 | XJ05 | China | 156808 | 87395 | 17853 | 25780 | CAA | CAA |
| **HUNAAS** | 8001 | XJ06 | China | 156817 | 87380 | 17853 | 25792 | CAA | CAA |
| **HUNAAS** | J02-17 | XJ07 | China | 156817 | 87380 | 17853 | 25792 | CAA | CAA |
| **HUNAAS** | SF11-1 | XJ09 | China | 156817 | 87380 | 17853 | 25792 | CAA | CAA |
| **HUNAAS** | SJ07-1 | XJ11 | China | 156811 | 87374 | 17853 | 25792 | CAA | CAA |
| **HUNAAS** | 0622-1-3-2-1 | XJ12 | China | 156792 | 87381 | 17853 | 25779 | CAA | CAA |
| **HUNAAS** | H1000-3-2-1-1-1 | XJ16 | China | 156817 | 87380 | 17853 | 25792 | CAA | CAA |
| **HUNAAS** | S105 | XJ17 | China | 156819 | 87380 | 17853 | 25793 | CAA | CAA |
| **HUNAAS** | SJ07-23 | XJ18 | China | 156819 | 87380 | 17853 | 25793 | CAA | CAA |
| **HUNAAS** | SJ09-79-1-1 | XJ20 | China | 156807 | 87370 | 17853 | 25792 | CAA | CAA |
| **HUNAAS** | LUO-2-2-2-1-1 | XJ21 | China | 156817 | 87380 | 17853 | 25792 | CAA | CAA |
| **HUNAAS** | SJ08-11-1 | XJ22 | China | 156819 | 87380 | 17853 | 25793 | CAA | CAA |
| **HUNAAS** | SJ05-43-1-1-1-1 | XJ23 | China | 156881 | 87380 | 17853 | 25824 | CAA | CAA |
| **HUNAAS** | XJ12-9-2 | XJ24 | China | 156793 | 87380 | 17853 | 25780 | CAA | CAA |
| **HUNAAS** | 0667-2-1-2-1-1 | YJ10 | China | 156811 | 87374 | 17853 | 25792 | CAA | CAA |
| **HUNAAS** | 9604 | YJ19 | China | 156817 | 87380 | 17853 | 25792 | CAA | CAA |
| **HUNAAS** | 9639 | YJ2 | China | 156817 | 87380 | 17853 | 25792 | CAA | CAA |
| **HUNAAS** | S51 | YJ21 | China | 156819 | 87380 | 17853 | 25793 | CAA | CAA |
| **HUNAAS** | 10H13-3-1-1-1-1 | YJ22 | China | 156807 | 87370 | 17853 | 25792 | CAA | CAA |
| **HUNAAS** | 6C-166 | YJ23 | China | 156817 | 87380 | 17853 | 25792 | CAA | CAA |
| **HUNAAS** | H1081-2 | YJ25 | China | 156793 | 87380 | 17853 | 25780 | CAA | CAA |
| **HUNAAS** | J02-82-1-1 | YJ26 | China | 156785 | 87374 | 17853 | 25779 | CAA | CAA |
| **HUNAAS** | J03-97 | YJ27 | China | 156797 | 87358 | 17853 | 25793 | CAA | CAA |
| **HUNAAS** | 06g19-1-1-1-1 | YJ28 | China | 156821 | 87384 | 17853 | 25792 | CAA | CAA |
| **HUNAAS** | S28 | YJ31 | China | 156794 | 87383 | 17853 | 25779 | CAA | CAA |
| **HUNAAS** | SJ09-115 | YJ32 | China | 156802 | 87389 | 17853 | 25780 | CAA | CAA |
| **HUNAAS** | SJ10-22 | YJ33 | China | 156797 | 87384 | 17853 | 25780 | CAA | CAA |
| **HUNAAS** | SJ10-32 | YJ34 | China | 156791 | 87380 | 17853 | 25779 | CAA | CAA |
| **HUNAAS** | 16YJ-35 | YJ35 | China | 156805 | 87368 | 17853 | 25792 | CAA | CAA |
| **HUNAAS** | 9703 | YJ36 | China | 156817 | 87380 | 17853 | 25792 | CAA | CAA |
| **HUNAAS** | 05S155-1-1 | YJ37 | China | 156811 | 87374 | 17853 | 25792 | CAA | CAA |
| **HUNAAS** | SJ09-48 | YJ38 | China | 156819 | 87380 | 17853 | 25793 | CAA | CAA |
| **HUNAAS** | SJ04-55-1-2-2-1-2 | YJ39 | China | 156817 | 87380 | 17853 | 25792 | CAA | CAA |
| **HUNAAS** | H1011-2-1 | YJ40 | China | 156817 | 87380 | 17853 | 25792 | CAA | CAA |
| **HUNAAS** | SJ09-135 | YJ41 | China | 156817 | 87380 | 17853 | 25792 | CAA | CAA |
| **HUNAAS** | NX880 | YJ49 | China | 156858 | 87421 | 17853 | 25792 | CAA | CAA |
| **HUNAAS** | A106 | YJ50 | China | 156817 | 87380 | 17853 | 25792 | CAA | CAA |
| **HUNAAS** | SJ15-19 | YJ52 | China | 156817 | 87380 | 17853 | 25792 | CAA | CAA |
| **HUNAAS** | SJ15-20 | YJ53 | China | 156831 | 87394 | 17853 | 25792 | CAA | CAA |
| **HUNAAS** | SJ09-19 | YJ54 | China | 156817 | 87380 | 17853 | 25792 | CAA | CAA |
| **HUNAAS** | J01-48-3-1 | YJ55 | China | 156727 | 87290 | 17853 | 25792 | CAA | CAA |
| **HUNAAS** | YJ58 | YJ58 | China | 156791 | 87380 | 17853 | 25779 | CAA | CAA |
| **HUNAAS** | LIU-40-1 | YJ59 | China | 156805 | 87368 | 17853 | 25792 | CAA | CAA |
| **HUNAAS** | J02-33 | YJ9 | China | 156817 | 87380 | 17853 | 25792 | CAA | CAA |
| **HUNAAS** | LJAS011 | YMH19 | Ethiopia | 156805 | 87368 | 17853 | 25792 | CAA | CAA |
| **HUNAAS** | LJBN002 | YMH32 | Benin | 156816 | 87379 | 17853 | 25792 | CAA | CAA |
| **HUNAAS** | LJAJ001 | YMH37 | Egypt | 156817 | 87380 | 17853 | 25792 | CAA | CAA |
| **HUNAAS** | LJAJ002 | YMH38 | Egypt | 157046 | 87342 | 17912 | 25896 | CAA | CG |
| **HUNAAS** | LJNR002 | YMH42 | Nigeria | 156817 | 87380 | 17853 | 25792 | CAA | CAA |
| **HUNAAS** | LJBX002 | YMH44 | Brazil | 156833 | 87396 | 17853 | 25792 | CAA | CAA |
| **HUNAAS** | LJBX005 | YMH47 | Brazil | 156817 | 87380 | 17853 | 25792 | CAA | CAA |
| **HUNAAS** | LJZB001 | YMH57 | Zambia | 156733 | 87296 | 17853 | 25792 | CAA | CAA |
| **HUNAAS** | LJSD014 | YMH64 | Sudan | 156817 | 87380 | 17853 | 25792 | CAA | CAA |
| **HUNAAS** | LJNF015 | YMH80 | South Africa | 156805 | 87368 | 17853 | 25792 | CAA | CAA |
| **HUNAAS** | LJNF017 | YMH82 | South Africa | 156730 | 87381 | 17853 | 25748 | CAA | CAA |
| **HUNAAS** | ZH-1 | ZH1 | China | 156983 | 87380 | 17853 | 25875 | CAA | CAA |
| **HUNAAS** | ZH-2 | ZH3 | China | 157029 | 87446 | 17853 | 25865 | CAA | CAA |

**Supplementary Table 1.** Continue.

| **Source** | **Identifier** | **Sample** | **Country** | **Length** | **LSC** | **SSC** | **IR** | **Label** | **Phylogeny** |
| --- | --- | --- | --- | --- | --- | --- | --- | --- | --- |
| **USDA-ARS** | Grif 9284 | PU0279 | Costa Rica | 157032 | 87407 | 17921 | 25852 | CAG | CG |
| **USDA-ARS** | Grif 9286 | PU0281 | Costa Rica | 156807 | 87379 | 17844 | 25792 | CAG | CAA |
| **USDA-ARS** | Grif 9331 | PU0324 | Costa Rica | 156954 | 87338 | 17912 | 25852 | CAG | CF |
| **USDA-ARS** | PI 310488 | PU1911 | Mexico | 157043 | 87404 | 17939 | 25850 | CAG | CAG |
| **USDA-ARS** | PI 439325 | PU3045 | Nicaragua | 157374 | 87406 | 17912 | 26028 | CAG | CG |
| **USDA-ARS** | PI 566812 | PU3635 | Mexico | 156753 | 87325 | 17844 | 25792 | CAG | CAA |
| **USDA-ARS** | PI 574547 | PU3639 | Mexico | 156957 | 87341 | 17912 | 25852 | CAG | CF |
| **USDA-ARS** | PI 593485 | PU3667 | Mexico | 157158 | 87366 | 17912 | 25940 | CAG | CG |
| **USDA-ARS** | PI 593486 | PU3668 | Mexico | 156784 | 87347 | 17853 | 25792 | CAG | CAA |
| **USDA-ARS** | PI 593490 | PU3672 | Mexico | 156733 | 87296 | 17853 | 25792 | CAG | CAA |
| **USDA-ARS** | PI 593493 | PU3675 | Mexico | 157422 | 87366 | 17912 | 26072 | CAG | CG |
| **USDA-ARS** | PI 593498 | PU3680 | Mexico | 157167 | 87375 | 17912 | 25940 | CAG | CG |
| **USDA-ARS** | PI 593499 | PU3681 | Mexico | 156982 | 87366 | 17912 | 25852 | CAG | CG |
| **USDA-ARS** | PI 593507 | PU3688 | Mexico | 157079 | 87375 | 17912 | 25896 | CAG | CG |
| **USDA-ARS** | PI 593517 | PU3695 | Mexico | 157158 | 87366 | 17912 | 25940 | CAG | CG |
| **USDA-ARS** | PI 593526 | PU3704 | Mexico | 157182 | 87390 | 17912 | 25940 | CAG | CG |
| **USDA-ARS** | PI 593543 | PU3720 | Mexico | 157431 | 87375 | 17912 | 26072 | CAG | CG |
| **USDA-ARS** | PI 593546 | PU3723 | Mexico | 156989 | 87351 | 17938 | 25850 | CAG | CAG |
| **USDA-ARS** | PI 593547 | PU3724 | Mexico | 156989 | 87351 | 17938 | 25850 | CAG | CAG |
| **USDA-ARS** | PI 593557 | PU3734 | Mexico | 156993 | 87354 | 17939 | 25850 | CAG | CAG |
| **USDA-ARS** | PI 593575 | PU3748 | Mexico | 156989 | 87351 | 17938 | 25850 | CAG | CAG |
| **USDA-ARS** | PI 593576 | PU3749 | Mexico | 156853 | 87310 | 17939 | 25802 | CAG | CAG |
| **USDA-ARS** | PI 593577 | PU3750 | Mexico | 156852 | 87309 | 17939 | 25802 | CAG | CAG |
| **USDA-ARS** | PI 593578 | PU3751 | Mexico | 156853 | 87310 | 17939 | 25802 | CAG | CAG |
| **USDA-ARS** | PI 631139 | PU3886 | Guatemala | 156954 | 87338 | 17912 | 25852 | CAG | CF |
| **USDA-ARS** | PI 631141 | PU3888 | Guatemala | 157641 | 87409 | 17912 | 26160 | CAG | CG |
| **USDA-ARS** | PI 632930 | PU3893 | Guatemala | 157465 | 87409 | 17912 | 26072 | CAG | CG |
| **USDA-ARS** | PI 632932 | PU3901 | Guatemala | 157224 | 87423 | 17921 | 25940 | CAG | CG |
| **USDA-ARS** | PI 674459 | PU4289 | Mexico | 157026 | 87322 | 17912 | 25896 | CAG | CG |
| **USDA-ARS** | PI 413669 | PU2762 | Colombia | 157150 | 87346 | 17974 | 25915 | CB | CB |
| **USDA-ARS** | PI 640883 | PU4186 | Mexico | 156817 | 87380 | 17853 | 25792 | CB | CAA |
| **HUNAAS** | A95, TC06842 | QT15 | China | 156932 | 87324 | 17866 | 25871 | CB | CB |
| **HUNAAS** | A115 | QT20 | China | 157108 | 87508 | 17850 | 25875 | CB | CB |
| **HUNAAS** | A87, C00302 | QT4 | China | 157145 | 87351 | 17974 | 25910 | CB | CB |
| **USDA-ARS** | PI 215699 | PU1190 | Peru | 156835 | 87398 | 17853 | 25792 | CBB | CAA |
| **USDA-ARS** | PI 238061 | PU1285 | Bolivia | 157149 | 87345 | 17974 | 25915 | CBB | CB |
| **USDA-ARS** | PI 260567 | PU1566 | Bolivia | 157150 | 87346 | 17974 | 25915 | CBB | CB |
| **USDA-ARS** | PI 281306 | PU1692 | Bolivia | 157149 | 87345 | 17974 | 25915 | CBB | CB |
| **USDA-ARS** | PI 439361 | PU3081 | Bolivia | 157149 | 87345 | 17974 | 25915 | CBB | CB |
| **USDA-ARS** | PI 439384 | PU3104 | Peru | 157630 | 87510 | 17930 | 26095 | CBB | CB |
| **USDA-ARS** | PI 439403 | PU3123 | Peru | 157150 | 87346 | 17974 | 25915 | CBB | CB |
| **USDA-ARS** | PI 439404 | PU3124 | Peru | 157150 | 87346 | 17974 | 25915 | CBB | CB |
| **USDA-ARS** | PI 633752 | PU3918 | Paraguay | 157057 | 87349 | 17974 | 25867 | CBB | CB |
| **USDA-ARS** | PI 639128 | PU3919 | Paraguay | 157237 | 87345 | 17974 | 25959 | CBB | CB |
| **USDA-ARS** | PI 585239 | PU0437 | Ecuador | 157568 | 87536 | 17930 | 26051 | CBPe | CB |
| **USDA-ARS** | PI 260488 | PU1513 | Bolivia | 157238 | 87346 | 17974 | 25959 | CBPe | CB |
| **USDA-ARS** | PI 260559 | PU1558 | Bolivia | 157150 | 87346 | 17974 | 25915 | CBPe | CB |
| **USDA-ARS** | PI 260561 | PU1560 | Bolivia | 157152 | 87346 | 17974 | 25916 | CBPe | CB |
| **USDA-ARS** | PI 260572 | PU1570 | Bolivia | 157150 | 87346 | 17974 | 25915 | CBPe | CB |
| **USDA-ARS** | PI 260576 | PU1573 | Bolivia | 157150 | 87346 | 17974 | 25915 | CBPe | CB |
| **USDA-ARS** | PI 260589 | PU1584 | Bolivia | 157150 | 87346 | 17974 | 25915 | CBPe | CB |
| **USDA-ARS** | PI 281308 | PU1694 | Bolivia | 157150 | 87346 | 17974 | 25915 | CBPe | CB |
| **USDA-ARS** | PI 281321 | PU1707 | Chile | 157150 | 87346 | 17974 | 25915 | CBPe | CB |
| **USDA-ARS** | PI 439363 | PU3083 | Bolivia | 157150 | 87346 | 17974 | 25915 | CBPe | CB |
| **USDA-ARS** | PI 439371 | PU3091 | Chile | 157150 | 87346 | 17974 | 25915 | CBPe | CB |
| **USDA-ARS** | PI 439385 | PU3105 | Peru | 157084 | 87346 | 17996 | 25871 | CBPe | CB |
| **USDA-ARS** | PI 439396 | PU3116 | Peru | 157128 | 87346 | 17952 | 25915 | CBPe | CB |
| **USDA-ARS** | PI 439398 | PU3118 | Peru | 157128 | 87346 | 17952 | 25915 | CBPe | CB |
| **USDA-ARS** | PI 439400 | PU3120 | Peru | 157062 | 87346 | 17974 | 25871 | CBPe | CB |
| **USDA-ARS** | PI 439406 | PU3126 | Peru | 157128 | 87346 | 17952 | 25915 | CBPe | CB |
| **USDA-ARS** | PI 439516 | PU3234 | Peru | 157150 | 87346 | 17974 | 25915 | CBPe | CB |
| **USDA-ARS** | PI 543178 | PU3541 | Bolivia | 157432 | 87346 | 17974 | 26056 | CBPe | CB |

**Supplementary Table 1.** Continue.

| **Source** | **Identifier** | **Sample** | **Country** | **Length** | **LSC** | **SSC** | **IR** | **Label** | **Phylogeny** |
| --- | --- | --- | --- | --- | --- | --- | --- | --- | --- |
| **USDA-ARS** | PI 593606 | PU3779 | Ecuador | 157152 | 87346 | 17974 | 25916 | CBPe | CB |
| **USDA-ARS** | PI 596055 | PU3866 | Bolivia | 157150 | 87346 | 17974 | 25915 | CBPe | CB |
| **USDA-ARS** | PI 260431 | PU1478 | Bolivia | 156866 | 87350 | 17898 | 25809 | CCha | CCha |
| **USDA-ARS** | PI 260438 | PU1484 | Bolivia | 157463 | 87319 | 17912 | 26116 | CCha | CG |
| **USDA-ARS** | PI 439413 | PU3133 | Argentina | 156765 | 87379 | 17844 | 25771 | CCha | CAA |
| **USDA-ARS** | PI 439414 | PU3134 | Argentina | 156732 | 87216 | 17898 | 25809 | CCha | CCha |
| **HUNAAS** | CXJ-B34 | CXJ1 | China | 156940 | 87324 | 17912 | 25852 | CC | CC |
| **HUNAAS** | CXJ-B147-1 | CXJ2 | China | 156753 | 87365 | 17840 | 25774 | CC | CC |
| **HUNAAS** | 19 | QT1 | China | 157112 | 87330 | 17912 | 25935 | CC | CC |
| **HUNAAS** | L2014-103 | QT23 | China | 156936 | 87330 | 17912 | 25847 | CC | CC |
| **HUNAAS** | A90, C04550 | QT6 | China | 156898 | 87293 | 17911 | 25847 | CC | CC |
| **HUNAAS** | A32 | QT7 | China | 156899 | 87293 | 17912 | 25847 | CC | CC |
| **HUNAAS** | CXJ-B156 | CXJ4 | China | 156704 | 87330 | 17840 | 25767 | CF | CF |
| **HUNAAS** | CXJ-B159 | CXJ6 | China | 156681 | 87335 | 17840 | 25753 | CF | CF |
| **HUNAAS** | CXJ-B160 | CXJ7 | China | 156616 | 87220 | 17840 | 25778 | CF | CF |
| **HUNAAS** | CXJ-A120 | CXJ74 | China | 156665 | 87267 | 17840 | 25779 | CF | CF |
| **HUNAAS** | CXJ-B161 | CXJ8 | China | 156685 | 87289 | 17840 | 25778 | CF | CF |
| **HUNAAS** | 14ML03-1 | QT22 | China | 156950 | 87344 | 17912 | 25847 | CF | CF |
| **HUNAAS** | CJ13-10 | QT27 | China | 156639 | 87259 | 17840 | 25770 | CF | CF |
| **HUNAAS** | LJSL002 | YMH23 | China | 156949 | 87343 | 17912 | 25847 | CF | CF |
| **HUNAAS** | LJSD010 | YMH60 | Sudan | 156954 | 87338 | 17912 | 25852 | CF | CF |
| **HUNAAS** | LJNF008 | YMH73 | South Africa | 156954 | 87338 | 17912 | 25852 | CF | CF |

HUNAAS: Institute of Vegetable Research, Hunan Academy of Agricultural Science, Changsha, China.

USDA-ARS: United States Department of Agriculture, Agricultural Research Service, USA.

CB: *Capsicum baccatum*; CBB: *Capsicum baccatum* var. *baccatum*; CBPe: *Capsicum* *baccatum* var. *pendulum*; CCha: *Capsicum chacoense*; CAA: *Capsicum annuum* var. *annuum*; CAG: *Capsicum* *annuum* var. *glabriusculum*; CC: *Capsicum chinense*; CF: *Capsicum frutescens*; CG: *Capsicum galapagoense*.

**Supplementary Table 2. The total annotated genes grouped by function and abbreviated accordingly.**

| **Functional group** | **Abbreviation** | **Genes** | | |
| --- | --- | --- | --- | --- |
| **Photosystem II** | ***psb*** | A | B | C |
|  |  | D | E | F |
|  |  | H | I | J |
|  |  | K | L | M |
|  |  | N | T | Z |
| **Ribosomal proteins (SSU)** | ***rps*** | 2 | 3 | 4 |
|  |  | 7^*3^ | 8 | 11 |
|  |  | 12^*2*3*4^ | 14 | 15 |
|  |  | 16^*1^ | 18 | 19 |
| **NADH dehydrogenase** | ***ndh*** | A^*1^ | B^*1*3^ | C |
|  |  | D | E | F |
|  |  | G | H | I |
|  |  | J | K | |
| **Ribosomal proteins (LSU)** | ***rpl*** | 2^*1*3^ | 14 | 16^*1^ |
|  |  | 20 | 22 | 23^*3^ |
|  |  | 32 | 33 | 36 |
| **Photosystem I** | ***psa*** | A | B | C |
|  |  | I | J | *ycf*3^*2^ |
|  |  | *ycf*4 | | |
| **Ribosomal RNAs** | ***rrna*** | 16^*3^ | 23^*3^ | 4.5^*3^ |
|  |  | 5^*3^ | | |
| **Cytochrome b/f complex** | ***pet*** | A | B^*1^ | D^*1^ |
|  |  | G | L | N |
| **ATP synthase** | ***atp*** | A | B | E |
|  |  | F^*1^ | H | I |
| **Other genes** | ***-*** | *acc*D | *ccs*A | *cem*A |
|  |  | *clp*P^*2^ | *mat*K |  |
| **RNA polymerase** | ***rpo*** | A | B | C1^*1^ |
|  |  | C2 | | |
| **Protein-coding (function unknown)** | ***ycf*** | *ycf*1^*3^ | *ycf*2^*3^ | *ycf*15^*3^ |
| **RubisCo large subunit** | ***-*** | *rbc*L | | |
| **Transfer RNAs** | ***trn*** | A-UGC^*1*3^ | C-GCA | D-GUC |
|  |  | E-UUC | F-GAA | G-UCC^*1^ |
|  |  | G-GCC | H-GUG | I-CAU^*3^ |
|  |  | I-GAU^*1*3^ | K-UUU^*1^ | L-UAA^*1^ |
|  |  | L-UAG | L-CAA^*3^ | fM-CAU |
|  |  | M-CAU | N-GUU^*3^ | P-UGG |
|  |  | Q-UUG | R-ACG^*3^ | R-UCU |
|  |  | S-GCU | S-GGA | S-UGA |
|  |  | T-GGU | T-UGU | V-UAC^*1^ |
|  |  | VGAC*3 | W-CCA | Y-GUA |

*1: Contain intron

*2: Contain two introns

*3: Two copies (in IRs) except for

*4: Trans-splicing gene

**Supplementary Table 3. Intra-species/variety diversity assessed for each species/variety separately, presented by number of non-InDel mutations (Eta), number of haplotypes (h) and its diversity (Hd), monomorphic and polymorphic SSR sites (frequency >1), InDel events (N=number of sites with at least one gap) and the trimmed plastome length.**

| **Species/**  **Variety** | **n*** | **Eta** | **h** | **Hd** | **Mono-**  **SSR** | **Poly-SSR unit**  **(region, length)** | **InDels**  **(N)** | **Trimmed plastome**  **length in bp** |
| --- | --- | --- | --- | --- | --- | --- | --- | --- |
| **CB** | 37 | 52 | 18 | 0.92 | 371 | AAT (*rbc*L-*acc*D, 26) | 30 (479) | 132,537 |
| **CCha** | 4 | 30 | 4 | 1.00 | 367 | 0 | 15 (205) | 132,185 |
| **CAG** | 10 | 13 | 6 | 0.86 | 365 | AT (*rps*16-*trn*Q, 30)  AAT (*ycf*1, 18) | 12 (177) | 132,318 |
| **CAA** | 238 | 19 | 24 | 0.81 | 360 | AT (*rps*16-*trn*Q, 101)  AAATT (*psa*A-*ycf*3, 82) | 28 (742) | 132,429 |
| **CC** | 9 | 21 | 7 | 0.94 | 361 | 0 | 21 (442) | 132,365 |
| **CF** | 15 | 36 | 8 | 0.82 | 360 | AT (*rps*16-*trn*Q, 58)  AAATT (*psa*A-*ycf*3, 57) | 15 (517) | 132,311 |
| **CG** | 30 | 52 | 28 | 0.92 | 364 | AT (*rps*16-trnQ, 38)  AAT (*ndh*C*-*trnV, 42)  AAT (*ycf*1, 29) | 79 (1029) | 132,611 |

*n= number of collapsed haplotypes + GenBank-deposited plastomes.

**Supplementary Table 4. Summary of the tandem repeats identified in pepper pan-plastome. Copy numbers were visually revised; copies failed to be recorded due to the intraspecies/variety variation were manually added and written in blue. Similarly, copies failed due to a SNP were manually added and written in green.**

| **loci** | **Unit size** | **CB** | **CCha** | **CAA** | **CAG** | **CC** | **CF** | **CG** |
| --- | --- | --- | --- | --- | --- | --- | --- | --- |
| *trn*K-*rps*16 | 13 | 2 | 2 | 2 | 2 | 2 | 2 | 2 |
| *rps*16-*trn*Q | 19 | 1 | 1 | 2 | 1 | 1 | 1 | 1 |
|  | 12 | 2 | 1 | 1 | 1 | 1 | 1 | 1 |
|  | 13 | 2 | **2** | 2 | 2 | 2 | 2 | 2 |
|  | 14 | 2 | 2 | 2 | 2 | 2 | 2 | 2 |
| *trn*S-*trn*G | 21 | 1 | 1 | 1 | 2 | 1 | 1 | 1 |
|  | 18 | 1 | 2 | 2 | 1 | 1 | 1 | 1 |
| *trn*G intron | 23 | 1 | 1 | 1 | 1 | 1 | 1 | 2 |
| *atp*H-*atp*I | 16 | 2 | 2 | 2 | 2 | 2 | 2 | 2 |
| *ycf*3 intron 1 | 15 | 2 | **3** | 2 | 2 | 2 | 2 | 2 |
| *trn*S-*rps*4 | 13 | 2 | 2 | 2 | 2 | 2 | 2 | 2 |
| *trn*T-*trn*L | 12 | 1 | 2 | 2 | 2 | 2 | 2 | 2 |
|  | 19 | 2 | 2 | 1 | 2 | 2 | 2 | 1 |
| *trn*L-*trn*F | 14 | 2 | 2 | 2 | 3 | **3** | **3** | **3** |
|  | 19 | 2 | 1 | **2** | **1** | 2 | 2 | **2** |
| *ndh*C-*trn*V | 14 | 2 | 2 | 2 | 2 | 2 | 2 | **2** |
| *trn*V-*trn*M | 18 | 1 | 1 | **2** | 2 | 2 | 2 | 2 |
| *trn*M-*atp*E | 13 | 2 | 2 | 2 | 2 | 2 | 2 | 2 |
| *rbc*L-*acc*D | 12 | **2** | 2 | 2 | 2 | 2 | 2 | 2 |
| *acc*D | 18 | **13** | **7** | **7** | **7** | **6** | **7** | **7** |
| *acc*D-*psa*I | 14 | 2 | **3** | 2 | **3** | 2 | 2 | 2 |
| *cem*A-*pet*A | 18 | 1 | 2 | 1 | 1 | 1 | 1 | 1 |
| *trn*P-*psa*J | 10 | 3 | 3 | 3 | 3 | 3 | 3 | 3 |
| *rps*18-*rpl*20 | 16 | 2 | 2 | 2 | 2 | 2 | 2 | **2** |
| *rps*12-*clp*P | 17 | 2 | 2 | 2 | 2 | 2 | 2 | **2** |
| *pet*D-*rpo*A | 14 | 2 | 1 | 1 | 1 | 1 | 1 | 1 |
| *rpl*23-*trn*I | 44 | **6** | 3 | **3** | 3 | 3 | **4** | **4** |
|  | 54 | 2 | 2 | 2 | 2 | 2 | 2 | 2 |
| *trn*I-*ycf*2 | 25 | **2** | 2 | **2** | **2** | **2** | **2** | **2** |
| *ycf*2 | 21 | 2 | 2 | **2** | 2 | 2 | 2 | **2** |

**Supplementary Table 5. List of the referenced plastomes retrieved from the GenBank database. For each accession, the deposited number, published species, variety if determined, accession name or number in the source database and its references are indicated. For each, the total plastome, long single copy (LSC), short single copy (SSC) and inverted repeats (IR) lengths are recorded. Species assignation based on the phylogenetic analysis is shown (Phylogeny).**

| **Accession number** | **Species** | **Variety** | **Material Source** | **Accession Name/No.** | **Length in bp** | **LSC** | **SSC** | **IR** | **Reference** | **Phylogeny** |
| --- | --- | --- | --- | --- | --- | --- | --- | --- | --- | --- |
| **JX270811** | *C. annuum* | ND | Monsanto, Korea | FS4401 | 156781 | 87366 | 17849 | 25783 | Jo et al. (2011) | CAA |
| **KJ619462** | *C. annuum* | *glabriusculum* | Wild accession, Mexico | Chiltepin | 156612 | 87177 | 17849 | 25793 | Zeng et al. (2016) | CG |
| **KR078311** | *C. annuum* | *glabriusculum* | NAC | IT158289 | 156817 | 87380 | 17853 | 25792 | Raveendar et al. (2015b) | CAA |
| **KR078312** | *C. frutescens* | - | NAC | IT158639 | 156817 | 87380 | 17853 | 25792 | Shim et al. (2016) | CAA |
| **KR078313** | *C. annuum* | *annuum* | NAC | Subicho (T216436) | 156878 | 87347 | 17929 | 25801 | Raveendar et al. (2015a) | CAG |
| **KR078314** | *C. baccatum* | *baccatum* | NAC | IT218961 | 157145 | 87351 | 17974 | 25910 | Kim et al. (2016) | CB |
| **KU041709** | *C. chinense* | - | PBI | ND | 156807 | 87290 | 17911 | 25803 | Park et al. (2016) ****** | CC |
| **KX913216** | *C. galapagoense* | - | NAC | - | 156959 | 87347 | 17918 | 25847 | Unpublished | CG |
| **KX913217** | *C. chinense* | - | NAC | IT247196 | 156936 | 87330 | 17912 | 25847 | Raveendar et al. (2017) ****** | CC |
| **KX913218** | *C. chacoense* | - | NAC | - | 156995 | 87379 | 17898 | 25859 | Unpublished | CCha |
| **KX913219** | *C. tovarii* | - | NAC | - | 156816 | 87379 | 17853 | 25792 | Shin et al. (2015) | CAA |
| **KX913220** | *C. eximium* | - | NAC | - | 156947 | 87341 | 17912 | 25847 | Unpublished | CF |
| **MH559320** | *C. baccatum* | *pendulum* | CGN | CGN21512 | 157144 | 87351 | 17973 | 25910 | D'Agostino et al. (2018) ****** | CB |
| **MH559321** | *C. chinense* | - | CGN | CGN22099 | 156858 | 87288 | 17860 | 25855 |  | CC |
| **MH559322** | *C. galapagoense* | - | CGN | CGN22208 | 157029 | 87366 | 17941 | 25861 |  | CA |
| **MH559323** | *C. annuum* | ND | IPK | CAP319 | 156842 | 87380 | 17960 | 25751 |  | CAA |
| **MH559324** | *C. baccatum* | *baccatum* | CGN | CGN23261 | 157053 | 87350 | 17973 | 25865 |  | CB |
| **MH559325** | *C. pubescens* | - | CGN | CGN22108 | 157390 | 87688 | 17928 | 25887 |  | - |
| **MH559326** | *C. frutescens* | - | CGN | CGN22779 | 156836 | 87359 | 17911 | 25783 |  | CG |
| **MH559327** | *C. annuum* | ND | CGN | CGN21526 | 157052 | 87380 | 17882 | 25895 |  | CAA |
| **MH559328** | *C. chacoense* | - | CGN | CGN22084 | 156841 | 87346 | 17893 | 25801 |  | CCha |
| **MH559329** | *C. annuum* | ND | IPK | CAP1546 | 156872 | 87341 | 17917 | 25807 |  | CG |
| **MH559330** | *C. baccatum* | *praetermissum** | CGN | CGN20805 | 157056 | 87351 | 17973 | 25866 |  | CB |

NAC: National Agrobiodiversity Center, Korea.

CGN: Centre for Genetic Resources germplasm bank, The Netherlands.

IPK: IPK Gatersleben germplasm bank, Germany.

PBI: Pepper and Breeding Institute, Suwon, Korea.

ND: Not determined / (-): not available.

* Considered a Variety of CB on GenBank database (<https://www.ncbi.nlm.nih.gov/nuccore/NC_039695.1>), but indicated as a species in the accession reference (D’Agostino et al., 2018).

** Phyloplastome analysis was performed.

**Supplementary Table 6.** Primer pair list used in the validation steps. The SNP based markers (*acc*D-*rbc*L, *rps*16-*trn*Q and *ycf*3-*trn*S) the confirmation on the LSC-IR boundary and the tandem repeat-based marker (rpl23-trnI).

| **Primer Name** | **Target *locus*** | **5'-Sequence-3'** | **Length** | **%GC** | **Tm ℃** |
| --- | --- | --- | --- | --- | --- |
| **ar_F** | *acc*D-*rbc*L | CAGTGGACGTTTTGGATAAG | 20 | 45 | 54.3 |
| **ar_R** |  | GAGTTTTCTTGCCCCCTATT | 20 | 45 | 55.3 |
| **rt_F** | *rps*16-*trn*Q | GTTGCTTTCTACCACATCGT | 20 | 45 | 56.1 |
| **rt_R** |  | GGAATGTGATTGTTGTTTCTGA | 22 | 36.4 | 55.2 |
| **yt_F** | *ycf*3-*trn*S | ATTTATACGTGATCTAGGCATAG | 23 | 34.8 | 53.4 |
| **yt_R** |  | GAATAGCGAGTCAGTCATAAC | 21 | 42.9 | 54 |
| **LR_F** | LSC-IR boundary | CATAGGAACGTCCGCGGATT | 20 | 55 | 60.2 |
| **LR_R** |  | GTCGGACAAGTGGGGAATGT | 20 | 55 | 60 |
| **90K_F** | *rpl*23-*trn*I | CTTTCCGGAAGTCGATGACT | 20 | 52.4 | 59.8 |
| **90K_R** |  | ATCCCCCTAAGCATCCATG | 20 | 50 | 60 |
